# Parsing variability in borderline personality disorder: a meta-analysis of neuroimaging studies

**DOI:** 10.1038/s41398-021-01446-z

**Published:** 2021-05-24

**Authors:** Giorgia Degasperi, Ioana Alina Cristea, Elisa Di Rosa, Cristiano Costa, Claudio Gentili

**Affiliations:** 1 Department of General Psychology, University of Padova, Padova, Italy; 2 Department of Brain and Behavioral Sciences, University of Pavia, Pavia, Italy; 3 IRCCS Mondino Foundation, Pavia, Italy; 4 Padova Neuroscience Center, University of Padova, Padova, Italy

**Keywords:** Neuroscience, Human behaviour, Psychiatric disorders

## Abstract

Though a plethora of functional magnetic resonance imaging (fMRI) studies explored the neurobiological underpinnings of borderline personality disorder (BPD), findings across different tasks were divergent. We conducted a systematic review and activation likelihood estimation (ALE) meta-analysis on the fMRI studies conducted in BPD patients compared to healthy controls (HC). We systematically searched PubMed and PsychINFO from inception until July 9th 2020 using combinations of database-specific terms like ‘fMRI’, ‘Neuroimaging’, ‘borderline’. Eligible studies employed task-based fMRI of the brain in participants of any age diagnosed with BPD compared to HC, during any behavioral task and providing a direct contrast between the groups. From 762 entries, we inspected 92 reports full-texts and included 52 studies (describing 54 experiments). Across all experiments, the HC > BPD and BPD > HC meta-analyses did not yield any cluster of significant convergence of differences. Analyses restricted to studies of emotion processing revealed two significant clusters of activation in the bilateral hippocampal/amygdala complex and anterior cingulate for the BPD > HC meta-analysis. Fail-safe N and single study sensitivity analysis suggested significant findings were not robust. For the subgroup of emotional processing experiments, on a restricted number of experiments providing results for each group separately, another meta-analysis method (difference of convergence) showed a significant cluster in the insula/inferior frontal gyrus for the HC > BPD contrast. No consistent pattern of alteration in brain activity for BPD was evidenced suggesting substantial heterogeneity of processes and populations studied. A pattern of amygdala dysfunction emerged across emotion processing tasks, indicating a potential pathophysiological mechanism that could be transdiagnostic.

## Introduction

According to the Diagnostic and Statistical Manual of Mental Disorders 5th edition (DSM 5), Borderline personality disorder (BPD) is characterized by a pervasive pattern of instability referred to interpersonal relationship, self-image and affects together with marked impulsivity and emotional dysregulation^1^. The disorder has a considerable prevalence (5.9% lifetime^2^) and is associated with significant and widespread impairment of patients’ lives^2–5^. A plethora of neuroimaging studies^6^, most of which employed functional Magnetic Resonance Imaging (fMRI), attempted to delineate the neurobiological underpinnings of BPD. However, findings across different types of tasks were divergent. For example, some studies showed activation increased in the amygdala^7,8^, insula^9^, occipital, frontal and temporal areas^10,11^, while others reported decreased activation in both frontal and temporal regions^12^, cingulate cortex and nucleus accumbens^13^. Yet other studies found no significant differences in activation between BPD and healthy subjects (HC)^14^. These contradictory findings could be due to methodological aspects related to differences in the processes studied (i.e, emotion processing, theory of mind, cognitive functions), paradigms or stimuli used, but also use of small samples, Region of Interest (ROI) analysis or uncorrected statistics. Conversely, heterogeneous findings could be indicative of “real” heterogeneity among BPD patient populations.

Hence, we conducted a systematic review and activation likelihood estimation (ALE) meta-analysis on the fMRI studies conducted in BPD patients compared to healthy controls. The meta-analytical technique considers nuclei of activation reported in single experiments as spatial probability distributions centered at the coordinate itself. These distributions are then used for the generation of a brain map representing the likelihood of activation of each candidate location^15^. An earlier meta-analysis on 19 studies^16^ focused solely on the contrast between negative and neutral stimuli and found higher convergence of differences in BPD as compared to HC in the left amygdalae and in the posterior cingulate, along with a blunted response of the bilateral dorsolateral prefrontal cortex. Here, we implemented a broader approach to BPD as we hypothesized that despite heterogeneity, a consistent pattern of dysfunction in BPD would nonetheless emerge, with more specific patterns observed in homogeneous subgroups. In particular, network analysis highlighted^17^ difficulties in emotional regulation as central features of BPD, while several reports underscored overlapping brain networks related to negative emotion processes and working memory^18^ or even a casual effect of difficulties in emotional regulation on other cognitive functions such mentalizing^19^ and working memory^20^. Thus, we expected that associated neurobiological dysfunctions would also impact other mental functions and hence emerge consistently across studies, despite the use of different tasks or evaluation domains.

## Methods

### Study selection

**Fig. 1:**
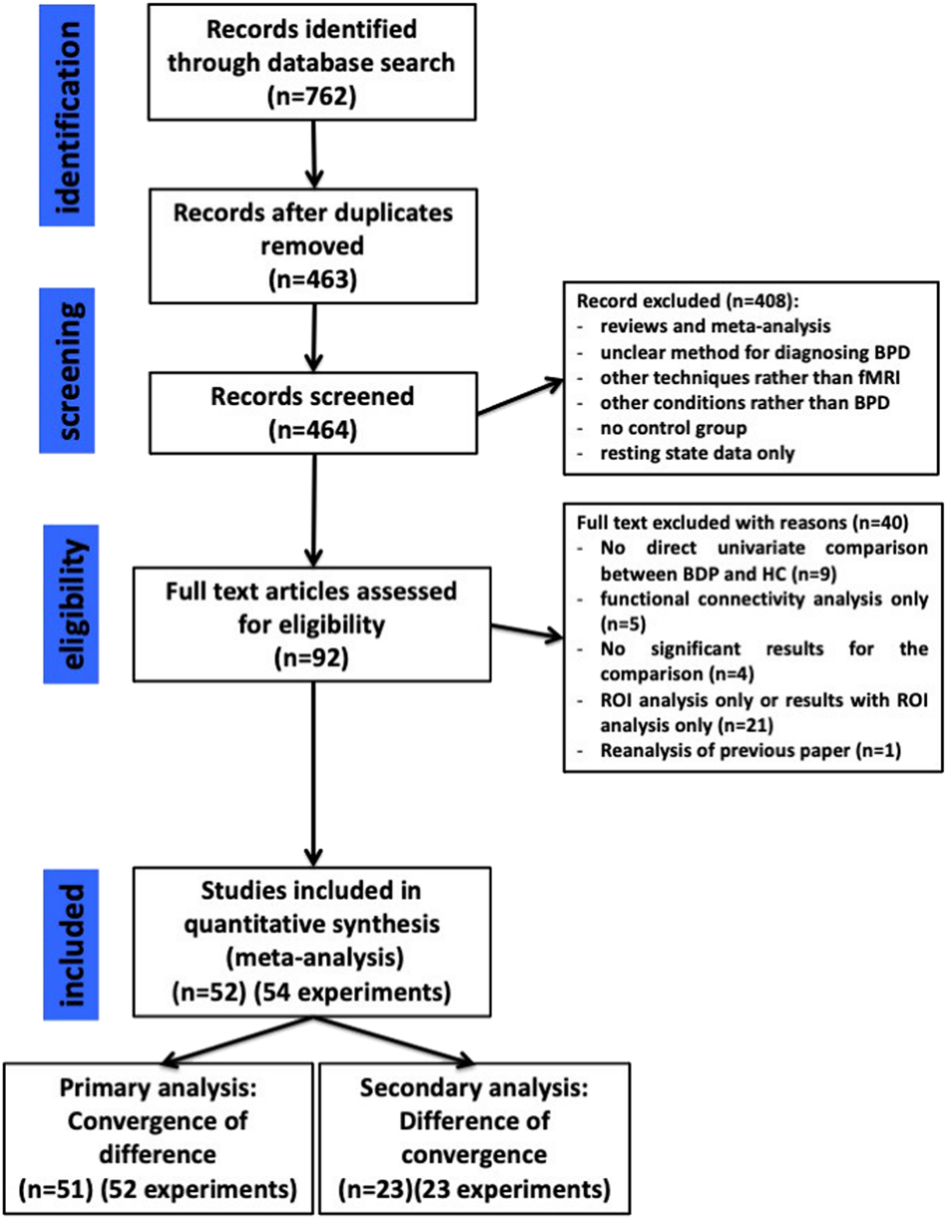
PRISMA diagram for the systematic research. BPD borderline personality disorder, HC healthy controls, ROI region of Interest.

The study was pre-registered on PROSPERO repository (CRD42019121856) and is reported following the PRISMA guidelines^21^ (see Supplementary Material for PRISMA checklist). We conducted systematic searches in PubMed and PsychINFO from inception until 9th of July 2020. We used combinations of database-specific terms as ‘fMRI’, ‘borderline’, ‘Neuroimaging’ (see Fig. 1 and Supplementary Material for the exact search string). Eligible studies (1) employed task-based fMRI of the brain in (2) participants of any age diagnosed with BPD according to the DSM IV, IV-TR or 5, based on diagnostic interviews, with or without comorbid disorders, (3) compared to a matched healthy control group (HC), during (4) any behavioral task using the same experimental paradigm was used for both BPD and HC, and had to include (5) a direct univariate comparison of brain activation between BPD and HC (i.e., BPD > BPD and/or BPD > HC), for which (6) 3D coordinates of peak activations in stereotactic space of the Montreal Neurological Institute (MNI) or Talairach were reported, and (7) whole-brain (i.e., not just Region of Interest/ROI) analysis were employed. There were no restrictions regarding receipt of any kind of treatment, past or current. For multiple reports on overlapping samples, only one (i.e., the one with the largest sample) was included. Reviews, meta-analyses and case-studies were excluded. Studies in English and Italian were considered eligible. Two authors (GD, CG) independently screened and selected studies.

### Data extraction

From each report, we extracted the following information: (1) participant mean age and gender (number of male and female participants); (2) comorbidities; (3) concurrent treatments; (5) type of task (e.g., passive or active tasks; task involving impulsivity control; emotional or cognitive tasks) and stimuli (e.g., faces, scripts, images, words); (6) coordinates for direct comparison of brain activation between BPD and HC; (7) where available, coordinates for the activations within each single group (BPD and HC). Data were extracted independently by two researchers (EdR, GD).

### Risk of Bias

The Risk of Bias (RoB) of included studies were evaluated with a modified version of the Newcastle-Ottawa scale (NOS)^22^, (mNOS) adapted to fMRI data^23^ (See supplementary material for details).

### ALE meta-analysis

Stereotactic coordinates for the ALE meta-analysis were extracted from the studies. The ALE algorithm was used as implemented in GingerALE 3.02 software^24^. We used the correction proposed by Turkeltaub and colleagues^15^, as implemented in GingerALE to control for multiple comparisons on the same dataset. Sample size for each experiment were used to calculate the Full‐Width Half‐Maximum (FWHM) of the Gaussian function used to blur the foci. Coordinates in the MNI 152 standard space were converted into the Talairach space using the GingerALE foci converter tool.

Two approaches can be employed in an ALE meta-analysis of two groups. The first (“convergence of activation differences”) uses coordinates from the contrast ‘patients vs. controls’ (i.e., patients > controls and controls > patients). The second (“differences in convergence”) pools the activation reported within each group separately, and subsequently computes a contrast between the resultant ALE-maps. We performed both for each of the two contrasts of interest (i.e., HC > BPD, BPD > HC) in each experiment.

We used convergence of activation differences as the primary analysis because it used data from all included studies. Statistical significance was assessed and corrected for multiple comparisons using a cluster-based method implemented in GingerALE: *p* < 0.001 cluster forming threshold, *p* < 0.05 cluster corrected FWE and *N* = 2000 permutations.

To check the robustness of the findings we performed a pooled analysis combining coordinates across BPD > HC and HC > BPD. This analysis reflects a more adequate summary of group differences because at the single study level, differences in analysis approaches and control conditions may have influenced the direction of reported group differences. To explore heterogeneity, we also conducted post-hoc sensitivity analyses by assembling more homogeneous subgroups of similar studies, based on extracted study characteristics such as type of task (e.g., passive or active tasks), type of stimuli (e.g., International Affective Picture System - IAPS) or type of domain evaluated (e.g., memory, impulsivity, emotion).

As additional robustness checks for analyses that produced significant findings, we conducted a “Fail-Safe N” analysis adapted for ALE meta-analysis^25^ to evaluate the potential publication bias, and a “leave-one-out” analysis to assess the impact of single studies on the results.

For the secondary analysis (differences in convergence), we computed separate meta-analyses for activations of controls and BPD and then contrasted them in a differences of convergence analysis. For the single group meta-analysis, we used the same parameters as in the primary analysis. To compute the differences of convergence, we used an uncorrected *p* value < 0.05, *N* = 10.000 permutations and a cluster threshold of 100 mm^3^.

Potential differences between the two methods could be attributed to the different number of included studies rather than to genuine discrepancies between methods. We tested for this hypothesis in sensitivity analyses, in which the primary and secondary methods were limited to the experiments reporting coordinates for the same type of contrasts for both single- and between groups results. (Fig. 1, Supplementary Methods).

Across all analyses, we set the minimum number of studies to 17. According to previous simulations, meta-analyses with less than 17 studies are likely to have insufficient power to detect smaller effects, increasing the risk that results are driven by single/few experiments^26,27^.

## Results

### Study selection

The search produced 762 entries (463 after duplicate removal), 371 of which were excluded based on the abstract, leaving 92 reports for full-text inspection. From these, 40 reports were further excluded due to (1) lack of direct univariate comparison between BPD and HC (*n* = 9); (2) comparison restricted to functional connectivity analysis (*n* = 5); (3) non-significant results for the comparison (*n* = 4); (4) ROI only reported (*n* = 21); (5) re-analyses of previous, already included, studies or paper reported no new results (*n* = 1). A total of 52 articles (Table 1 and Supplementary Table S1) were included in the meta-analysis, as described in the PRISMA flow diagram (Fig. 1). The 52 articles described 56 experiments and we further excluded two experiments for lack of significant results for the primary or secondary analysis.

**Table 1:** Characteristics of the included studied including, number of participants, gender and mean age of participants, comorbidities, concurrent treatments, type of tasks and type of stimuli used.

Study	Borderline personality disorder					Healthy controls			Task & stimuli	Type of stimuli
	N	M/F	Age (SD)	Comorbidity	Medication	N	M/F	Age (SD)		
Aguilar-Ortiz, 2019	67	3/64	31.54 (7.13)	?	AD, MS, AP	67	3/64	32.5 (9.68)	N-back task	Letters
Beblo, 2006	20	0/20	31.3 (8)	AvPD, Depr PD, PPD, P-APD, OCPD, DPD, PTSD, MDD, PD, PH, BN, OCD, DYS, GAD, HYP	SSRI, TAD, AP, b-block, BDZ, acamprosate	21	0/21	32.6 (7.8)	Recall of unresolved and resolved negative events	Individual cue words
Beeney, 2016	17	0/17	35.51 (10.84)	Aff D, Anx D, PTSD, AAD, SFD, ED	AD, AP, MS, BDZ	21	0/21	33.33 (9.81)	Personality trait evaluation	
Bertsch, 2019	15	15/0	27.9 (9.1)	Aff D, Anx D, PTSD, SAD, ED, APD, Sub D, SFD,	–	25	0/25	29.8 (5.6)	Social approach-avoidance task	Ekman and Friesen, KDEF, A&R, JACFEE
Brown, 2017	14	0/14	23.6 (4.1)	MDD, ED, Anx D, PTSD	AD, MS	17	0/17	23.2 (4.4)	Cyberball game	Virtual ball-tossing game
Buchheim, 2008	11	0/11	27.8 (6.7)	MDD, Anx D, PD, SFD, DD, PH, DA, AAD	AP, SSRI, Li	17	0/17	28.4 (7.5)	AAP	AAP
Cullen, 2016	12	0/12	25.17 (4.67)	?	AEP	12	0/12	24.17 (4.63)	Passive viewing of overt and covert emotional faces	Ekman and Friesen
Doell et al., 2020	21	0/21	27.43 (5.22)	MDD, BP, Anx D, PTSD, SAD, PD, GAD, ADHD; ED	SSRI, SNRI, AP, BDZ	24	0/24	24.71 (5.50)	Modified version of the monetary and social incentive delay task	KDEF
Domsalla, 2014	20	0/20	29.2 (7.5)	PTSD, PD, SAD, Spec P, OCD, BN, AN, Sub D, MDD, BP	–	20	0/20	28.7 (7.8)	Cyberball game	Virtual ball-tossing game
Dudas, 2017	14	0/14	36,3	?	PD	14	0/14	29,6	Emotion induction task	IAPS
Fertuck, 2019	16	0/16	25.94 (5.47)	PD, Spec P, GAD, SAD, PPD, OCPD, DPD, AvPD, ED, Sub D, MDD	–	17	0/17	23.71 (3.35)	Trustworthiness-Fear facial appraisal task	NimStim
Frick, 2012	21	0/21	27.14 (7.48)	PD, Spec P, GAD, PTSD, SAD, MDD, ED, PPD, STPD, OCPD, APD, NPD, P-APD, Sub D	–	20	0/20	24.80 (5.23)	Reading the mind in the eye’s test (RMET)	Original pictures of eye gazes
Gottlich et al., 2020	19	0/19	26.5 (5.8)	MDD, SAD, PD, PTSD, OCD, ADHD, ED, other personality disorders	SSRI, SNRI, TAI, AP, AEP, methylphenidate	22	0/22	26.4 (4.6)	Script-driven-imagery paradigm	Standardized scripts
Guitart-Masip, 2009	10	5/5	31.3 (9.47)	?	–	10	5/5	31.2 (9.05)	Emotional discrimination task	Ekman and Friesen series
Hazlett, 2012	33	13/20	31.6 (9.1)	?	–	32	12/20	32.8 (9.7)	Emotion recognition task	IAPS
Herbort, 2016	21	0/21	25.67 (5.98)	MDD, ED, AAD, DA, PTSD, SAD, AvPD, HPD	–	23	0/23	25.78 (5.75)	Categorial monetary incentive delay	
Herpertz, 2001	6	0/6	26.2 (8.1)	?	–	6	0/6	27.2 (4.5)	Passive viewing of emotion-inducing pictures	IAPS
Herpertz, 2017 (female sample)	33	0/33	26.19	Aff D, Anx D, PTSD, SFD, ED, AjD, APD, AvPD, Sub D		30	0/30	27.69 (6.38)	Script-driven-imagery paradigm	Standardized scripts
Herpertz, 2017 (male sample)	23	23/0	30.65 (8.75)	Aff D, Anx D, PTSD, ED, AjD, APD, AvPD, Sub D		26	26/0	31.09 (6.65)	Script-driven-imagery paradigm	Standardized scripts
Holtmann, 2013	16	0/16	25.56 (4.70)	PTSD, Sub D, PD, SAD, OCD, BN, AvPD, DPD, OCPD, HPD, MDD	–	24	0/24	26.83 (5.35)	Modified Eriksen Flanker task (emotional/neutral distractors)	Neutral or fearful faces
Homan, 2017	18	7/11	36.2 (11.1)	?	?	17	7/10	37.4 (12.4)	Social judgment task	Behavioral ad situational scenarios
King-Casas, 2008	55	4/51	32.9 (8.5)	?	?	38	1/37	31.3 (9.5)	Economic exchange game	Money exchange
Koenigsberg, 2009 (a)	18	10/8	32.6 (10.4)	GAD, PTSD, PPD, APD, NPD, DPD, HPD, OCPD, STPD, MDD, PD, ED	–	16	9/7	31.8 (7.7)	Distance from or look emotion-inducing pictures	IAPS
Koenigsberg, 2009 (b)	19	7/12	34.9 (11.1)	BD-II, PTSD, PPD, AvPD, APD, STPD, OCPD, HPD, NPD, MDD		17	8/9	31.2 (10.6)	Passive viewing of emotion-inducing pictures	IAPS
Koenigsberg, 2014	19	11/8	31.9 (9.9)	NPD, PPD, OCPD, APD, DPD, GAD, BED, DYS, OCD, Spec P, MDD, Sub D	–	25	13/12	28.1 (6.9)	Rating of novel and repeated emotion-inducing pictures	IAPS and EPS
Krauch, 2018 (adolescents)	20	0/20	16.35 (0.88)	Aff D, Anx D, PTSD, ED, Sub D	SSRI, Atypical AD	20	0/20	15.85 (0.81)	Script-driven imagery paradigm	
Kraus, 2010	11	0/11	25.64 (3.83)	DYS, PD, AP, SAD, PTSD, Spec P, AvPD, PPD, BD-II, MDD, AN, BN, AAD, Sub D	–	10	0/10	25.6 (5.23)	Script-driven imagery	Standard script describing self-injury behavior
Krause-Utz, 2017 (dissociation)	17	0/17	27.41 (6.20)	PTSD, MDD, DYS, PD, SAD, Spec P, OCD, ED, AAD, SD, DA		18	0/18	29.61 (8.61)	EWMT	Uppercase letters and IAPS as distractors
Krause-Utz, 2017 (neutral)	12	0/12	25.17 (6.21)	PTSD, MDD, DYS, PD, SAD, Spec P, OCD, ED, AAD, SD, DA	–	18	0/18	29.61 (8.61)	EWMT	
Lamers, 2019	20	0/20	25.95 (6.94)	PTSD, PTSD and SAD, SAD, SAD and AP, Spec P, OCD, BN	AD, AP	20	0/20	26.90 (9.43)	Passive viewing of emotion-inducing stimuli	Movie clips of faces
Lang, 2012	14	0/14	27.21 (7.66)	PD, PH, GAD, SAD, DA, AAD, MDD, ED, PPD, STPD, SzPD, OCPD, APD, NPD	–	15	0/14	24.73 (5.64)	Up- and down-regulation to negative scripts	
Malejko, 2018 (a)	15	0/15	23.33 (1.07)	MDD, PTSD, DYS	AD (SSRI, SNRI, MAOIs)	15	0/15	23.27 (1.11)	Somatosensory unpleasant stimulation	Electrical
Malejko, 2018 (b)	15	0/15	23.3 (4.13)	PTSD, DYS, PTSD	AD (SSRI, SNRI, MAOIs)	17	0/17	23.1 (4.26)	Cyberball paradigm	Virtual ball-tossing game
Mensebach, 2009	18	0/18	31.94 (8.13)	PTSD, MDD, DYS, PD, AP, OCD, AP with PD, PH, BN, SD	SSRI, TAD, AP, b-block, BDZ, acamprosate	18	0/18	32.94 (8.33)	EMR/SMR	Words
Mier, 2013	13	9/4	28.15 (7.06)	MDD, Sub D, GAD, PTSD, AjD, MDCE	AD, AP, MS, BDZ	13	8/5	30.46 (4.29)	Neutral face processing, Emotion recognition, Affective ToM	Emotional facial expression
Minzenberg, 2007	12	5/7	30.3 (8)	OCD, PTSD, PD, PPD, STPD, APD, NPD, HPD, AvPD	–	12	6/6	30.7 (10)	Gender discrimination	Ekman and Friesen set (modified)
Mortensen, 2016	14	0/14	30.1 (6.7)	–	–	14	0/14	28.3 (7.4)	Posner task	
Nicol, 2015	20	17/3	35.8 (8.6)	MDD, BP, ED, PTSD, OCD	AD, AP	16	14/2	34.8 (9.6)	Gender recognition task	Ekman series of emotional faces
Niedtfeld, 2010	20	0/20	30.50 (8.30)	?	–	23	0/23	27.13 (8.26)	Picture and temperature stimulus presentation	IAPS and warm stimulus
Peters, 2018	13	0/13	21.23 (3.30)	?	SSRI	16	0/16	21.81 (4.02)	Provocation task, Directed rumination task	
Scherpiet, 2014	18	0/18	28.44 (8.50)	Sub D, ED, MDD, ADHD, PTSD	AD, AP, MS, BDZ, zolpidem	18	0/18	28.89 (7.11)	Emotional anticipation task	IAPS
Scherpiet, 2015	19	0/19	31.11 (6.29)	MDD, MDD with Sub D, ADHD, ADHD with Sub D, Sub D, PTSD, ED	AD, AP, MS, BDZ, pramipexole	19	0/19	29.37 (4.48)	Mindful introspection, Cognitive self-reflection	
Schmahl, 2006	12	0/12	28.67 (5.88)	MDD, DYS, PD, PD with AP, AP, PTSD, SAD, BDD, AN, OCD, SzPD, PPD, OCPD, AvPD, DA, Sub D	–	12	0/12	27.67 (6.83)	Heat stimulation	Fixed temperature and individual temperature stimuli
Schnell, 2007 (a)	6	0/6	23.7 (4.8)	BN, DYS	–	6	0/6	23.4 (5.0)	Passive viewing of emotion-inducing pictures	IAPS and similar photographic images
Schnell, 2007 (b)	14	0/14	28 (5.77)	ED, PG, PTSD, ADD	–	14	0/14	28. 4 (5.21)	Passive viewing of emotion-inducing pictures	TAT and IAPS as control stimuli
Schulze, 2011	15	0/15	27.60 (7.85)	PTSD, OCD, PD with AP, AP, BN, DD, DYS, AAD, Sub D	–	15	0/15	24.53 (2.85)	Delayed reappraisal paradigm	IAPS and supplementary similar pictures
Silbersweig, 2007	16	15/1	31.25 (range 19-50)	PD, SAD, Spec P, PTSD, OCD, GAD, SD, AAD, DA	AD, AP, MS, BDZ	14	10/4	23.8 (18-31)	Emotional linguistic go/no-go task	Negative, positive and neutral words
Sosic-Vasic, 2019	20	0/20	24.9 (5.26)	MDD, DYS, PD, AP, SAD, GAD, PTSD, BN	AD (SSRI, SNRI, NaSSA, TAD)	20	0/20	26.05 (5.96)	Passive viewing of emotion-inducing stimuli	Stylized scenes showing reaction to loss
van Schie, 2019	26	0/26	30.46 (9.2)	MDD, DYS, PD, AP, SAD, Spec P, GAD, PTSD, ADHD, AAD, D, APD, PPD	AD (SSRI, SNRI)	32	0/32	28.12 (9.8)	Social feedback task	Evaluative words
van Zutphen, 2017	55	0/55	30.80 (8.78)	MDD, DYS, BP, GAD, PD, AP, PD with AP, SAD, Spec P, OCD, PTSD, SFD, ED, Sub D, IED, AvPD, DPD, OCPD, P-APD, Depr PD, PPD, STPD, SzPD	AD, AP, MS, BDZ	42	0/42	28.33 (10.50)	Emotion regulation paradigm	IAPS and erotic pictures
van Zutphen, 2019	53	0/53	31.02 (8.77)	MDD, DYS, BP, GAD, PD, AP, PD with AP, SAD, Spec P, OCD, PTSD, SFD, ED, Sub D, IED, AvPD, DPD, OCPD, P-APD, Depr PD, PPD, STPD, SzPD	AD, AP, MS, BDZ	34	0/34	29.44 (11.31)	Affective go/no-go task	IAPS and erotic picture
Wingenfeld, 2009	20	14/6	29.75 (13.2)	PTSD, MDD, DYS, BN, GAD, SPD	SSRI, MAOIs, AP	20	14/6	29.45 (12.4)	Individual emotional Stroop task	General words and individual negative words from individual report
Winter, 2015 (neutral)	19	0/19	28.05 (7.82)	MDD, DYS, PD, SAD, Spec P, OCD, PTSD		19	0/19	28.74 (8.07)	Emotional stroop task	Aachener emotional word list
Wrege, 2019	39	30/9	27.5 (8.2)	Depr PD, Anx D, SD, ED, SFD, PTSD	AD, MS, AP	29	25/4	25.7 (6.0)	Cyberball paradigm	Virtual ball-tossing game

*Comorbidity?* comorbidity was not declared, *–* comorbidity was an exclusion criteria, *AAD* alchool abuse disorder, *ADD* attention deficit disorder, *ADHD* attention deficit hyperactivity disorder, *Aff D* affective disorder, *AjD* adjustment disorder, *AN* anorexia nervosa, *Anx D* anxiety disorder, *AP* agoraphobia, *APD* antisocial personality disorder, *AvPD* avoidant personality disorder, *BD* bipolar disorder, *BDD* body dysmorphic disorder, *BED* binge eating disorder, *BN* bulimia nervosa, *DA* drug abuse, *DD* dissociative disorder, *Depr PD* depressive personality disorder, *DPD* dependent personality disorder, *DYS* dyshymia, *ED* eating disorder, *GAD* generalized anxiety disorder, *HPD* histrionic personality disorder, *HYP* hypochondria, *IED* intermitted explosive disorder, *MDCE* mixed disorder of conduct and emotion, *MDD* major depressive disorder, *NPD* narcisistic personality disorder, *OCD* obsessive compulsive disorder, *OCPD* obsessive compulsive personality disorder, *P-APD* passive-aggressive personality disorder, *PD* panic disorder, *PG* pathological gambling, *PH* phobia, *PPD* paranoid personality disorder, *PTSD* post-traumatic stress disorder, *SAD* social anxiety disorder, *SD* somatization disorder, *SFD* somatoform disorder, *Spec P* specific phobia, *SPD* somatization pain disorder, *STPD* schizotypal personality disorder, *Sub D* substance use disorder, *SzPD* schizoid personality disorder, *Medication?* the medication status of the subjects was not described, – ongoing medication was an exclusion criteria, *AD* antidepressants, *AEP* anti-epileptic drugs, *AP* antipsychotics, *Atypical AD* atypical antidepressants, *b-block* beta blockers, *BDZ* benzodiazepines, *Li* lithium, *MAOIs* monoamine oxidase inhibitors, *MS* mood stabilizers, *NaSSA* noradrenergic and specific serotonergic antidepressants, *PD* psychotropic drugs, *SNRI* serotonin-norepinephrine reuptake inhibitors, *SSRI* serotonin reuptake inhibitors, *TAD* tryciclic antidepressant, *Task & stimuli* type of stimuli, *AAP* the adult attachment projective, *A&R* the AR face database, Martinez and Benavente (1998), *Affective ToM* affective theory of mind, *EMR/SMR* episodic and semantic memory retrieval task, *EPS* empathy picture system, *EWMT* emotional working memory task, *IAPS* international affective picture system, *JCFEE* Matsumoto and Ekman’s Japanese and Caucasian facial expressions of emotion, *KDEF* The Karolinska directed emotional faces, Lundqvist et al. (1998), *NimStim* NimStim face database, Tottenham et al. (2009), *TAT* thematic apperception test.

### Characteristics of included studies

The 54 experiments included 2084 subjects (1104 BPD and 1100 HC). All studies performed whole-brain analyses. For the primary analysis we used 52 experiments: 24 reported both contrasts HC > BPD and BPD > HC, seven the HC > BPD contrast only, whereas 21 the BPD > HC contrast only. Twenty-three studies also reported single group analyses (Fig. 1 and Supplementary Table S1): 21 for both HC and BPD, one for HC only and two for BPD only. A complete description of the studies including type of task, stimuli, presence of comorbidity and medications status is presented in the Table 1). Based on these characteristics, we assembled more homogenous groups for sensitivity analyses. We conducted analyses restricted to studies (1) using only active or (2) passive tasks; (3) employing a task related to emotion processing (generation, recognition, regulation) (4) restricted to unmedicated patients.

### Risk of bias

According to our mNOS scale scores no study presents a high risk of bias, eleven have low risk while the great majority (41) have an intermediate risk (Supplementary Table S2 and Supplementary Fig. S1). The full description of the study quality is described in the Supplementary Material.

### Primary analysis: convergence of differences

For the voxel-wise whole-brain analysis 51 reports (52 experiments) were considered. For the HC > BPD meta-analysis we included 31 experiments and obtained a minimum cluster size of 624 (MCS) mm³, while for the BPD > HC meta-analysis we included 45 experiments and obtained an MCS of 752 mm³. Across both ALE meta-analyses, we did not find any cluster of significant convergence.

### Sensitivity analyses for primary analysis

**Fig. 2:**
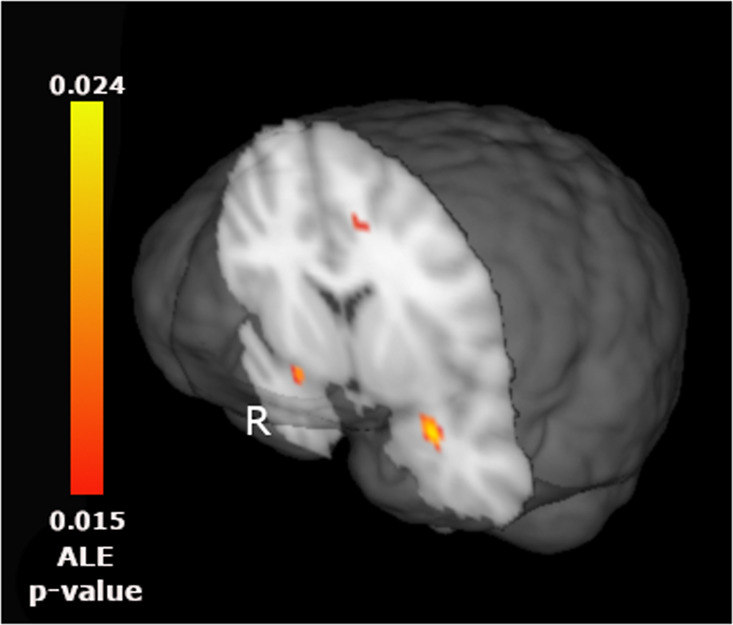
Sensitivity analysis for the primary analysis (convergence of difference) including studies with Emotional task (without studies on impulsivity) as stimuli for the BPD > HC meta-analysis. Results are cluster-wise corrected (uncorrected *p* value < 0.001, cluster-wise corrected *p* value < 0.05). R right side, ALE-*p* value activation likelihood estimation probability.

In analysis restricted to studies of emotion processing (42 experiments) for the contrast BPD > HC (34 experiments, MCS 656 mm^3^), we found significant clusters in the two amygdalae along with the anterior cingulate (ACC)/middle frontal gyrus (MFG) (Table 2 and Fig. 2). For the contrast HC > BPD (24 Experiments, MCS 680) we did not find any significant cluster.

**Table 2:** Sensitivity analysis for the primary analysis (convergence of difference) including studies on emotions (without considering impulsivity and reward).

Contrast	Hemisphere	Region	BA	Center of mass			Peak			Peak ALE *p* value	Volume (mm³)
				x	y	z	x	y	z		
BPD > HC	L	Amygdala		29.9	2.1	−17.8	30	0	−20	0.023	784
	R	Amygdala		−28	2.2	−17.4	−28	−4	−18	0.024	776
	L	MFG		−11.7	26	32	−12	34	26	0.017	736
		MFG					−8	28	32	0.017	
		ACC					−12	20	34	0.017	
		ACC					−14	14	38	0.016	
		MFG					−14	32	34	0.015	

Results are cluster-wise corrected (uncorrected *p* value < 0.001, cluster-wise corrected *p* value < 0.05).

*HC* healthy controls, *BPD* Borderline Personality Disorders, *MFG* Middle Frontal Gyrus, *ACC* Anterior Cingulate Cortex.

We were interested in the distinct pattern of activation related to emotional regulation, however only six studies specifically addressed emotional modulation in BPD^28–33^, too few for a meta-analysis. As impulsivity is a key feature of BPD^17^ with specific brain correlates only partially overlapping with the broader emotional circuits, we considered these studies separately. However, only four^13,34–36^ studies examined impulsivity.

Analyses restricted to studies of unmedicated individuals showed largely similar results of the emotional processing meta-analysis (Supplementary Material, Supplementary Fig. S2 and Supplementary Table S3). Another sensitivity analysis restricted to studies using active tasks did not produce significant results. There were too few studies using passive tasks (12 for BPD > HC and 2 for HC > BPD) to run a meta-analysis. Finally, the pooled analysis using both the HC > BPD and the BPD > HC contrasts in the same meta-analysis did not show significant results (Supplementary Material).

The leave-one-out procedure conducted on the subgroup of emotion processing studies (the only ones with statistically significant findings), showed that the clusters in the right amygdala remained significant 29 over 34 times, the cluster in the left amygdala, 27 times and the ACC/MFG cluster 24 times. Notably, excluding one particular study^37^ led to no significant cluster of convergence, excluding three studies^8,38,39^ led to only one significant cluster, and excluding other 13 studies preserved two out of three significant clusters (Table 3). The Fail-Safe N analysis showed that the addition of only two studies rendered the ACC/MFG cluster no longer significant. Similarly, three hypothetical studies would make the left amygdala finding null. Adding 33 studies would result in no remaining significant cluster of convergence (Supplementary Fig. S3).

**Table 3:** Leave-one-out (LOO) analysis.

	Contribution to clusters			LOO			No. of remaining clusters
	R amy	L amy	ACC	R amy	L amy	ACC	
Frick, et al. 2012	x						0
Minzenberg, et al. 2007	x		x		x		1
Hazlett, et al. 2012	x				x		1
Schnell, et al. 2007 (b)	x			x			1
Koenigsberg, et al. 2009 (a)	x				x	x	2
Cullen, et al. 2016		x		x		x	2
Niedtfeld, et al. 2010		x		x		x	2
Herpertz, et al. 2017		x		x		x	2
Holtmann, et al. 2013			x		x	x	2
Gottlich, et al. 2020			x	x	x		2
Herpertz, et al. 2001				x		x	2
Mier, et al. 2013				x		x	2
Dudas, et al. 2017				x	x		2
Schulze, et al. 2011				x	x		2
Scherpiet, et al. 2014				x	x		2
Schnell, et al. 2007 (a)				x	x		2
Wrege, et al. 2019				x	x		2
Domsalla, et al. 2014				x	x	x	3
Peters, et al. 2018				x	x	x	3
Guitart-Masip, 2009				x	x	x	3
Koenigsberg, et al. 2009 (b)				x	x	x	3
Kraus, et al. 2010				x	x	x	3
Winter, et al. 2015				x	x	x	3
Krause-Utz, et al. 2018				x	x	x	3
Koenigsberg, et al. 2014				x	x	x	3
Buchheim, et al., 2008				x	x	x	3
Beblo, et al. 2006				x	x	x	3
Brown, et al. 2017				x	x	x	3
Sosic-Vasic, et al. 2019				x	x	x	3
Malejko, et al. 2018(b)				x	x	x	3
Lamers, et al. 2019				x	x	x	3
Bertsch, et al. 2019				x	x	x	3
Doell, et al. 2020				x	x	x	3
van Zutphen, et al. 2017				x	x	x	3
Total				28	27	23	

The columns 2–4 indicates whether each article contributes directly to the cluster according to the gingerALE program; columns 5–7 indicates the effect of each article removal on the results in the three significant clusters; column 8 indicates the number of clusters remaining after leave each article out.

### Secondary analysis: difference in convergences

Twenty-three experiments reported coordinates for single group analyses (23 for HC and 22 for BPD) (Supplementary Methods and Supplementary Table S1). Results for the meta-analysis within each group are reported in the Supplement (Supplementary Table S4 and Supplementary Fig. S4). No significant clusters were identified for the BPD > HC contrast. For the HC > BPD a significant cluster of difference in convergences between the two groups was highlighted in the right insula/ inferior frontal gyrus (IFG) (Supplementary Table S5 and Supplementary Fig. S5).

The number of eligible studies for all sensitivity analyses less than 17. Nevertheless, we computed exploratory sensitivity analyses (considering only emotion processing and respectively only active task experiments), which showed no significant results (see Supplementary Results) (Supplementary Tables S6 and S7 and Supplementary Fig. S6).

## Discussion

We report, to our knowledge, the first meta-analysis comprising all fMRI neuroimaging studies for borderline personality disorder. The main findings underscore the substantial heterogeneity of this literature, in reference to the processes studied (e,g., emotion^40,41^, impulsivity^36,42^, attention and working memory^43^), as well as study populations (e.g., concomitant medication, comorbidities). Importantly, though impulsivity and emotional dysregulation are cardinal symptoms of BPD, they were assessed in few studies (four and six respectively). However, methodological differences can represent other important sources of heterogeneity. A variety of behavioral tasks were used, and it is likely that some were more reliable and robust in measuring the target processes than others, as demonstrated in a large-scale analysis of self-regulation measures^44^. Moreover, analytic pipelines including pre-processing, choices in data analysis like the type of multiple comparison corrections employed most likely diverged between studies, with direct consequences on the threshold for identifying statistical findings^45–48^. The relationship between analytic choices and reporting statistically significant findings is particularly relevant for ALE meta-analyses, which exclusively rely on these results and cannot consider non-significant one^49^.

### Convergence of differences

Analyses restricted to more homogeneous subgroups highlighted significant clusters of convergence in the primary analysis. Specifically, we found dysfunctional pattern in the two amygdalae and ACC/MFG across emotion processing tasks. Of note, analyses limited to studies with non-medicated patients also resulted in a significant cluster of convergence in the right hippocamus/amygdala. Nonetheless, all the studies on unmedicated participants also investigated emotional processing, which might account for the overlap in results.

The role of the amygdala complex in emotional processing is well-established. Emotional responses were associated with activations of the amygdalae^50,51^, while effective emotional regulation strategies reduce amygdala reactivity^52,53^. Furthermore, anxiety disorders and mood disorders^23,54–58^ are characterized by amygdala hyperreactivity, normalized by effective pharmacological and psychological treatments^59,60^. Thus, our results support the role of amygdala dysfunction as a transdiagnostic mechanism, present in BPD, similarly to other disorders. It was hypothesized that behavioral alterations in emotional regulation and impulsivity in BPD rely on abnormal amygdala activity or a dysfunctional interaction between amygdala and prefrontal cortex, in line with findings on studies for emotional processing^61,62^. As a consistent finding in BPD and as marker of emotional dysfunction on BPD, amygdala altered activity was proposed as a potential predictor of treatment response as well as a target for neurofeedback interventions^63,64^. Finally, the development of new drug treatments has been hypothesized and tested considering their known action over amygdala activity^65^. A similar transdiagnostic role could be attributed to ACC, though this region was less frequently reported in prior studies. Activation in this region has been reported while retrieving emotionally negative life events^66^, during social exclusion^67^ and while processing negative emotions^61,68^ more generally. All these processes are affected in BPD, as well as in other emotional disorders.

Our findings partially confirm those of an earlier meta-analysis by Schulze and colleagues^16^. Divergences may stem from the fact the current meta-analysis included almost twice as many studies, (34 vs. 19 for the emotional processing sub-group) reflecting the large number of studies published in the last 4 years. Other sources of discrepancy include different inclusion criteria, meta-analytic approaches (as Schulze and colleagues used Anisotropic Effect Size Signed Differential Mapping^69^, which allows the combination of coordinates and unthresholded maps) and contrasts selected in the analysis.

Despite the considerably larger pool of studies, the robustness of the emotional processing findings is limited. Fail-Safe N analysis showed that as few as three additional null studies would render the clusters in the ACC and in the left amygdala no longer significant. Conversely, for the right amygdala, 33 studies with null findings would render the result not statistically significant. The Fail-Safe N is a proxy for potential publication bias (i.e., studies that were conducted, but remained unpublished, “in the file-drawer”, because of negative or null results). According to the adaptation of this method for ALE meta-analysis^25^, for findings to be robust to publication bias, at least twice the number of included studies should be necessary to make them non-significant. In the present report, additional evidence for publication bias comes from the high number of studies that could not be included due to not reporting significant findings or conducting only ROI analyses. Thus, the hypothetical null studies suggested by the Fail-Safe N might not even have to be searched in the file-drawer, but already published. Solutions for clarifying similar issues in the neuroimaging literature include access to unthresholded maps, for example by posting them in public repositories, so as to allow studies with null or negative findings to be included in neuroimaging meta-analyses. Likewise, pre-registration of ROI analyses^49^, by guaranteeing these analyses were not contingent to non-significant whole-brain results, could support their inclusion in meta-analyses. The results of the leave-one-out analysis for the emotional processing subgroup similarly point to limited robustness. The three significant clusters were maintained in only 13 iterations, but with the exclusion of a single study^37^, no results remain significant. This suggests that findings are heavily influenced by a few individual studies.

Finally, we failed to find convergence for the HC > BPD. While this contrast was reported in only 24 experiments, it is interesting to note that only four studies specifically discussed the dysfunction of emotional processing. As dysfunctions in emotional modulation are believed to be caused by a lack of activation in the prefrontal cortex in BPD, we speculate that a higher number of studies on emotional regulation would result in significant HC > BPD convergence.

### Difference in convergence

The contrast HC > BPD resulted in a significant cluster of difference of convergence in the right insula/IFG complex. The activation of the inferior frontal gyrus has been often described for tasks related to emotional regulation and modulation^18^, thus is not surprising how control subjects showed a higher convergence in this region as compared to BPD. This might be due to a general lack of IFG activity in BPD or to a higher heterogeneity of brain activations in this group.

### Convergence of difference or difference in convergence?

According to Muller and colleagues the difference between the convergence of difference and difference in convergence approaches are mirrored by the research question one wants to answer^26^. The former aims at identifying common difference between groups across the experiments, whereas the latter focuses to the convergence within the groups across the experiments and then to their possible differences. We confirm that these two approaches produce divergent findings, as previously shown^70^. Comparison of the two methods restricted to experiments reporting the data required to run both resulted in a significant cluster in the anterior cingulate for convergence of difference, and another in the left insula for difference of convergence, for the HC > BPD contrast, suggesting that divergences between the two methods are not imputable to different numbers of studies.

The discrepancy between the two approaches is probably rooted into the ALE meta-analysis method of taking only coordinates of significant clusters. We proposed that the convergence of differences method is more feasible for contrasting groups, because it generally relies on a larger number of studies and is usually more concordant with findings from individual studies^70^. In our work, differences might also be related to the relatively poor robustness of the primary analysis findings. For example, Hazellet and colleagues^8^, a study that if excluded resulted into two of the three significant clusters becoming non-significant, did not provide single group results, so could not be used for the difference in convergence analysis.

However, the two methods could also be viewed as complementary. In a recent ALE meta-analysis on depression the heterogeneity of the depressed patients was proposed as a possible reason for the lack of significant convergence^71^: the depressed groups of each study might be too neurobiologically different among each other thus the convergence of difference did not produce consistent results. Specifically, while we found no significant clusters with the difference of convergence method, we did find clusters of convergence for both HC and BPD with the convergence of difference method. This suggests that despite task-related heterogeneity, a common pattern of activation within BPD patients can be identified. We suggest that this result is a hint to the fact that the heterogeneity of BPD population is similar to the one of healthy controls although a more formalized and quantitative approach to heterogeneity should be applied (e.g.,^72^) to confirm this speculation.

### Limitations

By design, ALE meta-analysis can only quantify convergence probabilities and not magnitude of activations. The method provides a probability of convergence, that is, concordance of statistically significant foci across experiments in terms of probability distributions centered at the each set of focus coordinates^24^. Moreover, coordinate based meta-analyses rely exclusively on coordinates for contrasts that reached statistical significance, as defined in each individual study. Consequently, these methodologies involve a significant loss information as compared to aggregation of fMRI unthresholded maps^73^ and cannot take into account publication bias^25^. Unfortunately, open sharing of unthresholded maps remains limited^74^. Another limitation is related to the considerable number of studies that were excluded due to reporting only on ROI analyses (*N* = 23). Though the ROI approach is widely employed in neuroimaging studies, particularly in small sample studies, it is associated with increased risk of false positives or overestimation of effects^49^. Moreover, its use is discouraged in coordinate based meta-analysis^23,26^. Unfortunately, requests for whole-brain results are often not honored^54^.

## Conclusion

A meta-analysis of neuroimaging studies identified a consistent, albeit not robust, pattern of activation for emotional processing. No other common pattern of convergence of activation emerged, probably owing to the high between-study heterogeneity, ranging from tasks, populations and analytic approaches. Our findings mirror those of another ALE meta-analysis on unipolar depression^71^. Though this meta-analysis included a large number of neuroimaging experiments (99), overall analyses across cognitive and emotional processing experiments, as well as subgroup analyses, revealed no consistent pattern of convergence. As possible causes for the lack of significant convergence of activation, the authors list differences among individual studies (such as the use of uncorrected inference procedures, differences in experimental design and contrasts, or heterogeneous clinical populations) and meta-analytic approaches (such as different inclusion and exclusion criteria or too liberal statistical inference methods). Though we did report a pattern of amygdala and ACC dysfunction in studies of emotion processing, it was not robust to possible publication bias and single study effects. Amygdala dysfunction might represent a promising pathophysiological mechanism, as a well target for novel therapeutic strategies^63–65^, though, conversely, it might also indicate a transdiagnostic marker of difficulties in emotional processing and regulation. Further studies may disentangle this aspect particularly evaluating emotional regulation difficulties both in BPD and across psychopathological domains with a single paradigm. Network analyses have shown how difficulties in emotional regulation are the most relevant core feature of BPD^17^, applying the same approach to neuroimaging data across psychiatric disorders may help to clarify the specificity of the present findings. Perhaps more importantly than conducting new studies, public sharing of unthresholded maps^75^ from already conducted ones would represent a great advance in the understanding of the neurobiology of BPD.

## Supplementary information

Supplementary material
