# Supplementary material for: Parsing variability in borderline personality disorder: a meta-analysis of neuroimaging studies
Source: Transl Psychiatry. 2021 May 24;11:314. doi: 10.1038/s41398-021-01446-z (PMC8144551; doi:10.1038/s41398-021-01446-z)
Supplement: Supplementary file 1 — Supplementary material [file 41398_2021_1446_MOESM1_ESM.docx]

**Supplementary material**

**S1. Supplementary Methods**

**S1.1. Search String:**

Search date 30th of July 2020

*Pubmed:*

(("magnetic resonance imaging"[MeSH Terms] OR ("magnetic"[All Fields] AND "resonance"[All Fields] AND "imaging"[All Fields]) OR "magnetic resonance imaging"[All Fields] OR "fmri"[All Fields]) OR ("magnetic resonance imaging"[MeSH Terms] OR ("magnetic"[All Fields] AND "resonance"[All Fields] AND "imaging"[All Fields]) OR "magnetic resonance imaging"[All Fields] OR ("functional"[All Fields] AND "magnetic"[All Fields] AND "resonance"[All Fields] AND "imaging"[All Fields]) OR "functional magnetic resonance imaging"[All Fields]) OR ("neuroimaging"[MeSH Terms] OR "neuroimaging"[All Fields] OR ("brain"[All Fields] AND "imaging"[All Fields]) OR "brain imaging"[All Fields]) OR ("neuroimaging"[MeSH Terms] OR "neuroimaging"[All Fields])) AND ("borderline personality disorder"[MeSH Terms] OR ("borderline"[All Fields] AND "personality"[All Fields] AND "disorder"[All Fields]) OR "borderline personality disorder"[All Fields] OR ("borderline"[All Fields] AND "personality"[All Fields]) OR "borderline personality"[All Fields]) 430 entries

*Psychinfo search:*

fmri or functional magnetic resonance imaging or brain imaging or neuroimaging (all fields) AND

borderline personality (all fields) 385 entries

**S1.2 Study quality evaluation:**

Risk of bias (RoB) was independently assessed by two authors (GD), EdR). Inter-rater agreement was measured with the kappa statistic, and disagreements were subsequently resolved by discussion between assessors and a third author (CC). Scores on the modified Newcastle-Ottawa scale (mNOS) range from 0 to 12, with 0 to 3 considered indicative of high risk, 4 to 7 as intermediate and 8 to 12 as low risk. The scale is composed of four domains: 1) the quality of sample selection, evaluated according to i) case definition, ii) representativeness, iii) selection of the control group, and iv) definition of controls; 2) the quality of reproducibility, evaluated considering whether the samples were comparable in terms of age and other variables considered in the study; 3) the quality of exposure, evaluate based on i) whether the two groups underwent the same experimental procedure, ii) whether drop-outs were reported, and iii) whether a behavioral effect was measured. Additionaly, a fourth domain to assess quality in data analysis has been introduced. Specifically, in this domain we evaluated i) whether the studies used a sufficient cluster-forming threshold (i.e., voxel-based uncorrected p-value) to compensate for false positive underestimation, as recently described ^1^, and ii) whether the authors used and described a valid correction method to avoid/reduce false positive results (for further information on NOPS scale, see also ^2^).

**S1.3 ALE meta-analyses**

All the sensitivity analyses for the primary and secondary analyses were performed with the same parameters. Therefore, for the convergence of difference (primary analysis) the following parameters were used: *p*<0.001 for cluster-forming uncorrected threshold and p<0.05 for cluster‐level inference, and N = 2000 permutations. In turn for the difference in convergence (secondary analysis), we used an uncorrected p < 0.05, N=10.000 permutations and a cluster threshold of 100 mm^3^.

**S2. Supplementary Results**

**S2.1. Characteristics of included studies**

We included 52 articles in our meta-analysis describing 54 experiments (table S1) ^3-54^.

Only one study included patients without comorbidities, while ten did not explicitly mention comorbidity as inclusion/exclusion criteria. The most frequent comorbidities reported in the remaining 41 experiments were mood and anxiety disorders. Twenty-two studies recruited unmedicated subjects, while two did not explicitly mention the state of medication use of the patients. In the remaining 27 studies, a vast selection of drugs was used, alone or in combination, including antidepressants and mood stabilizers as the most frequent. Due to the limited number of studies including participants without comorbidities, we did not run sensitivity analysis. However, we ran a sensitivity analysis on studies with unmedicated subjects only. Forty-one studies reported the use of active tasks (e.g., GO/No-GO), and 13 used passive tasks (e.g., images passive viewing). Forty-three (of these only one reported HC and BPD groups separately without the direct comparison between the two grousp) studies focused on emotional processing. Regarding stimuli, only ten studies used faces, while 14 used pictures from the International Affective Picture System (IAPS). A complete list of type of task and stimuli is presented in table 1.

**S2.2. Study Quality**

The overall Cohen kappa (Mean ±SD) was 0.88±0.10 ranging from 1 to 0.66. Consensus and Cohen kappa for each item of the mNOS were reported in Table S2 and Figure S1. The lower agreement was for preregistration (0.66) and definition of controls (0.77). Eleven studies were considered as low RoB, while the other 40 were rated as intermediate risk.

Forty-nine studies provided an adequate case definition, but in only twelve, the sample could be considered representative of the population. Fourteen studies provided an adequate selection of controls, and forty-one a satisfactory description of criteria used to select control participants. Regarding comparability, age and gender were matched between groups in 36 studies, whereas other variables (e.g., IQ) were controlled for in 37. In all studies, patients and controls underwent the same experimental procedure (as expected, given this was an inclusion criteria). Eighteen studies reported drop-outs, and 24 found between groups differences in behavioural tasks. Regarding statistical quality, the adoption of a cluster-forming threshold to control for false positive results in neuroimaging studies (p<0.001) ^1,55^ was used 22 studies. Thirty-seven studies reported the use of adequate methods to correct for multiple comparisons, and only in one the protocol was pre-registered.

**S3.3. Sensitivity analyses for the primary analysis (convergence of difference)**

Analyses restricted to active tasks involved 33 experiments for BPD>HC (MCS 600 mm³) and 29 for the HC>BPD (MCS 592 mm³) meta-analyses. No significant cluster was found for either. A limited number of experiments (12 for BPD>HC and 2 for HC>BPD) employed passive tasks, precluding a meaningful aggregation in a meta-analysis.

The sensitivity analysis on unmedicated patients yielded a significant cluster of convergence in the right hippocampal/amygdala complex for the BPD>HC contrast (21 experiments, MCS 568 mm^3^) (Table S3 and Figure S2), while the HC>BDP contrast was present in an insufficiently small number of experiments (15). Finally, the pooled analysis combining coordinates across BPD>HC and HC>BPD contrasts yielded no significant results.

**S3.4 Sensitivity analyses for the secondary analysis (difference in convergence)**

Analyses restricted to active tasks for HC (16 experiments, minimum cluster size 720 mm^3^) did not converge, so the comparison for HC vs. BPD could not be computed. For BPD (16 experiments, minimum cluster size 784 mm^3^), convergence was significant in the left inferior frontal gyrus/insula (table S4).

Analyses restricted to emotion processing studies showed significant convergence in the right inferior frontal gyrus (IFG) for BPD (15 experiments, minimum cluster size 696 mm^3^), and, respectively, in the bilateral inferior frontal gyrus and left amygdala for HC (15 experiments, minimum cluster size 672 mm^3^) (table S4). The difference of convergence between the two meta-analyses, however, did not yielded any significant cluster.

**S3.5 Comparison between the two meta-analytic methods**

We applied both techniques (convergence of difference and difference in convergence) on the pool of studies that reported both the between group (HC>BDP and BPD>HC) and the single groups analyses and in which identical contrasts of interest were investigated for both types of analysis (Table S6). We used the same parameters as in the respective primary and secondary analyses. The convergence of difference meta-analysis showed a single significant cluster in the anterior cingulate (15 experiments, MCS 616 mm3) for the HC>BPD contrast only (Figure S6a, Table S7). The difference of convergence highlighted a single cluster for the HC >BPD contrast, centered in the left IFG (Figure S6b, Table S7).

**S4. Supplementary Tables**

**Table S1**: list of studies entering each meta-analysis of the primary and secondary analyses and sensitivity analyses.

|  | **coordinates for single groups** | | **coordinates for contrasts** | |  |  |  |  |
| --- | --- | --- | --- | --- | --- | --- | --- | --- |
| **Study** | **HC** | **BPD** | **BPD > HC** | **HC >BPD** | **type** | **emotion** | **reward/ impulsivity** |  |
|  |  |  |  |  |  |  |  | |
| Aguilar-Ortiz, 2019 | x | x | x | x | active |  |  | |
| Beblo, 2006 |  | x | x |  | active | x |  | |
| Beeney, 2016 |  |  | x | x | active |  |  | |
| Bertsch, 2019 |  |  | x | x | active | x |  | |
| Brown, 2017 |  |  | x |  | passive | x |  | |
| Buchheim, 2008 | x | x | x | x | active | x |  | |
| Cullen, 2016 |  |  | x |  | passive | x |  | |
| Doell 2020 |  |  | x | x | active | x |  | |
| Domsalla, 2014 |  |  | x |  | passive | x |  | |
| Dudas, 2017 |  |  | x | x | active | x |  | |
| Fertuck, 2019 |  |  |  | x | active | x |  | |
| Frick, 2012 | x | x | x | x | active | x |  | |
| Gottlich 2020 |  |  | x | x | active | x |  | |
| Guitart-Masip, 2009 |  |  | x |  | active | x |  | |
| Hazlett, 2012 |  |  | x |  | active | x |  | |
| Herbort, 2016 | x | x |  | x | active |  | x | |
| Herpertz, 2001 | x | x | x |  | passive | x |  | |
| Herpertz, 2017 (female sample) |  |  |  | x | active | x |  | |
| Herpertz, 2017 (male sample) |  |  | x |  | active | x |  | |
| Holtmann, 2013 | x | x | x |  | active | x |  | |
| Homan, 2017 |  |  | x |  | active |  |  | |
| King-Casas, 2008 |  |  | x | x | active |  | x | |
| Koenigsberg, 2009 |  |  | x | x | active | x |  | |
| Koenigsberg, 2009 | x | x | x | x | active | x |  | |
| Koenigsberg, 2014 | x | x | x | x | active | x |  | |
| Krauch, 2018 (adolescents) |  |  |  | x | active | x |  | |
| Kraus, 2010 | x | x | x | x | active | x |  | |
| Krause-Utz, 2018 (dissociation) |  |  | x |  | active | x |  | |
| Krause-Utz, 2018 (neutral) |  |  | x |  | active | x |  | |
| Lamers, 2019 | x | x | x |  | passive | x |  | |
| Lang, 2012 | x | x |  | x | active | x |  | |
| Malejko, 2018(a) | x | x |  |  | passive | x |  | |
| Malejko, 2018(b) |  |  | x |  | passive | x |  | |
| Mensebach, 2009 | x | x | x |  | active |  |  | |
| Mier, 2013 | x | x | x | x | active | x |  | |
| Minzenberg, 2007 | x | x | x | x | active | x |  | |
| Mortensen, 2016 | x | x | x | x | active |  | x | |
| Nicol, 2015 |  |  |  | x | active | x |  | |
| Niedtfeld, 2010 |  |  | x | x | passive | x |  | |
| Peters, 2018 |  |  | x |  | active | x |  | |
| Scherpiet, 2014 | x | x | x |  | passive | x |  | |
| Scherpiet, 2015 | x | x | x | x | active |  |  | |
| Schmahl, 2006 | x | x | x | x | passive |  |  | |
| Schnell, 2007 (a) |  |  | x |  | passive | x |  | |
| Schnell, 2007 (b) | x | x | x |  | passive | x |  | |
| Schulze, 2011 | x | x | x | x | active | x |  | |
| Silbersweig, 2007 |  |  | x | x | active | x |  | |
| Sosic-Vasic, 2019 |  |  | x |  | passive | x |  | |
| van Schie, 2019 |  |  | x | x | active |  |  | |
| van Zutphen, 2017 |  |  | x | x | active | x |  | |
| van Zutphen, 2019 |  |  | x | x | active |  | x | |
| Wingenfeld, 2009 | x |  |  | x | active | x |  | |
| Winter, 2015 (neutral) |  | x | x | x | active | x |  | |
| Wrege, 2019 |  |  | x |  | active | x |  | |

*Note.* HC: healthy controls; BPD: Borderline Personality Disorders; IAPS: International Affective Picture System

**Table S2:** Study-level quality ratings and the kappa inter-rater agreement before reaching consensus

|  | **Selection** | | | | **Compartbility** | | **Exposure** | | | **Statistical analysis** | | |
| --- | --- | --- | --- | --- | --- | --- | --- | --- | --- | --- | --- | --- |
| **Study** | **adequate case definition** | **representativeness** | **controls** | **definition of controls** | **age and gender** | **other variables** | **same exposure** | **drop-out rate** | **behavioral manipulation check** | **p value > 0.001** | **false positive correction** | **Pre-Registration** |
| Aguilar-Ortiz, 2019 | 1 | 1 | 0 | 2 | 0 | 1 | 1 | 1 | 0 | 2 | 0 | 0 |
| Beblo, 2006 | 1 | 0 | 2 | 1 | 1 | 1 | 1 | 0 | 1 | 2 | 0 | 0 |
| Beeney, 2016 | 1 | 0 | 0 | 1 | 1 | 1 | 1 | 0 | 1 | 2 | 1 | 0 |
| Bertsch, 2019 | 1 | 0 | 1 | 1 | 0 | 1 | 1 | 1 | 0 | 2 | 1 | 0 |
| Brown, 2017 | 1 | 1 | 1 | 1 | 1 | 2 | 1 | 0 | 1 | 1 | 0 | 0 |
| Buchheim, 2008 | 1 | 0 | 0 | 2 | 1 | 1 | 1 | 1 | 1 | 1 | 0 | 0 |
| Cullen, 2016 | 1 | 0 | 1 | 1 | 1 | 1 | 1 | 0 | 0 | 2 | 1 | 0 |
| Doell 2020 | 1 | 0 | 0 | 1 | 0 | 1 | 1 | 0 | 1 | 1 | 0 | 0 |
| Domsalla, 2014 | 1 | 0 | 0 | 2 | 1 | 1 | 1 | 0 | 0 | 1 | 1 | 0 |
| Dudas, 2017 | 1 | 0 | 0 | 1 | 0 | 2 | 1 | 0 | 1 | 2 | 1 | 0 |
| Fertuck, 2019 | 1 | 0 | 1 | 1 | 0 | 1 | 1 | 1 | 1 | 2 | 1 | 0 |
| Frick, 2012 | 1 | 0 | 1 | 1 | 1 | 1 | 1 | 0 | 1 | 1 | 0 | 0 |
| Gottlich 2020 | 1 | 0 | 0 | 1 | 0 | 1 | 1 | 1 | 1 | 1 | 1 | 0 |
| Guitart-Masip, 2009 | 1 | 1 | 2 | 1 | 1 | 2 | 1 | 0 | 1 | 1 | 1 | 0 |
| Hazlett, 2012 | 1 | 1 | 2 | 1 | 1 | 1 | 1 | 0 | 1 | 2 | 0 | 0 |
| Herbort, 2016 | 1 | 0 | 2 | 1 | 1 | 1 | 1 | 0 | 0 | 1 | 1 | 0 |
| Herpertz, 2001 | 1 | 1 | 1 | 2 | 1 | 1 | 1 | 0 | 1 | 1 | 1 | 0 |
| Herpertz, 2017 | 1 | 0 | 1 | 2 | 1 | 1 | 1 | 0 | 0 | 0 | 1 | 0 |
| Holtmann, 2013 | 1 | 0 | 2 | 1 | 1 | 1 | 1 | 0 | 0 | 1 | 1 | 0 |
| Homan, 2017 | 1 | 2 | 2 | 1 | 1 | 2 | 1 | 1 | 0 | 1 | 1 | 0 |
| King-Casas, 2008 | 1 | 0 | 2 | 1 | 0 | 1 | 1 | 0 | 1 | 2 | 1 | 0 |
| Koenigsberg, 2009 | 1 | 1 | 1 | 1 | 1 | 2 | 1 | 0 | 0 | 0 | 0 | 0 |
| Koenigsberg, 2009 | 1 | 1 | 1 | 1 | 1 | 1 | 1 | 0 | 0 | 2 | 1 | 0 |
| Koenigsberg, 2014 | 1 | 1 | 1 | 1 | 1 | 2 | 1 | 1 | 1 | 2 | 1 | 0 |
| Krauch, 2018 | 1 | 0 | 2 | 1 | 1 | 1 | 1 | 0 | 0 | 1 | 1 | 0 |
| Kraus, 2010 | 1 | 0 | 0 | 1 | 1 | 1 | 1 | 1 | 0 | 2 | 1 | 0 |
| Krause-Utz, 2017 | 1 | 0 | 0 | 1 | 1 | 1 | 1 | 0 | 0 | 1 | 1 | 0 |
| Lamers, 2019 | 1 | 0 | 0 | 1 | 0 | 1 | 1 | 1 | 1 | 2 | 1 | 0 |
| Lang, 2012 | 1 | 0 | 1 | 1 | 1 | 1 | 1 | 0 | 0 | 1 | 1 | 0 |
| Malejko, 2018a | 1 | 0 | 1 | 2 | 0 | 1 | 1 | 0 | 1 | 2 | 1 | 0 |
| Malejko, 2018b | 1 | 0 | 0 | 1 | 0 | 1 | 1 | 1 | 0 | 1 | 0 | 0 |
| Mensebach, 2009 | 1 | 0 | 2 | 1 | 1 | 1 | 1 | 1 | 2 | 1 | 0 | 0 |
| Mier, 2013 | 2 | 2 | 2 | 2 | 1 | 1 | 1 | 0 | 0 | 1 | 0 | 0 |
| Minzenberg, 2007 | 1 | 1 | 1 | 1 | 1 | 1 | 1 | 1 | 0 | 0 | 0 | 0 |
| Mortensen, 2016 | 1 | 0 | 0 | 2 | 1 | 1 | 1 | 0 | 1 | 0 | 1 | 0 |
| Nicol, 2015 | 1 | 1 | 0 | 1 | 1 | 1 | 1 | 0 | 1 | 0 | 2 | 0 |
| Niedtfeld, 2010 | 1 | 0 | 0 | 1 | 1 | 2 | 1 | 1 | 0 | 2 | 1 | 0 |
| Peters, 2018 | 1 | 0 | 1 | 1 | 1 | 1 | 1 | 0 | 2 | 2 | 1 | 0 |
| Scherpiet, 2014 | 1 | 0 | 2 | 2 | 1 | 2 | 1 | 1 | 0 | 2 | 1 | 0 |
| Scherpiet, 2015 | 2 | 0 | 2 | 2 | 1 | 2 | 1 | 1 | 2 | 2 | 1 | 0 |
| Schmahl, 2006 | 1 | 0 | 2 | 1 | 1 | 2 | 1 | 0 | 1 | 2 | 1 | 0 |
| Schnell, 2007 | 1 | 0 | 0 | 1 | 1 | 1 | 1 | 0 | 2 | 0 | 1 | 0 |
| Schnell, 2007 | 0 | 0 | 2 | 1 | 2 | 1 | 1 | 0 | 1 | 1 | 0 | 0 |
| Schulze, 2011 | 1 | 0 | 0 | 2 | 1 | 1 | 1 | 1 | 0 | 1 | 1 | 0 |
| Silbersweig, 2007 | 1 | 2 | 2 | 1 | 0 | 2 | 1 | 0 | 1 | 0 | 0 | 0 |
| Sosic-Vasic, 2019 | 1 | 0 | 0 | 1 | 0 | 2 | 1 | 0 | 2 | 1 | 1 | 0 |
| van Schie, 2019 | 1 | 0 | 0 | 1 | 0 | 0 | 1 | 1 | 1 | 2 | 1 | 0 |
| van Zutphen, 2017 | 1 | 0 | 0 | 1 | 0 | 1 | 1 | 2 | 1 | 2 | 1 | 0 |
| van Zutphen, 2019 | 1 | 0 | 0 | 1 | 1 | 0 | 1 | 1 | 0 | 0 | 1 | 0 |
| Wingenfeld, 2009 | 1 | 1 | 0 | 1 | 1 | 1 | 1 | 0 | 0 | 1 | 0 | 0 |
| Winter, 2015 | 1 | 0 | 2 | 1 | 1 | 1 | 1 | 0 | 1 | 1 | 1 | 0 |
| Wrege, 2019 | 1 | 1 | 0 | 1 | 0 | 0 | 1 | 0 | 1 | 2 | 1 | 1 |
| **Agreement** | **1** | **0.91** | **0.80** | **0.76** | **1** | **0.91** | **1** | **0.96** | **0.84** | **0.88** | **0.82** | **0.66** |

**Table S3:** Sensitivity analysis for the primary analysis (convergence of difference) including studies with unmedicated patients only.

|  |  |  |  | Centre of mass | | | Peak | | | |  | |  | |  |
| --- | --- | --- | --- | --- | --- | --- | --- | --- | --- | --- | --- | --- | --- | --- | --- |
| Contrast | **Hemisphere** | **Region** | **BA** | **x** | **y** | **z** | | **x** | **y** | **z** | | **Peak ALE p-value** | | **Volume (mm³)** | |
| BPD > HC | R | Parahippocampal gyrus/Amygdala |  | 30.3 | 2.5 | -17.4 | | 30 | 0 | -20 | | 0.022 | | 776 | |

*Note.* HC: healthy controls, BPD: Borderline Personality Disorder. Results are cluster- wise corrected (uncorrected p-value < 0.001, cluster-wise corrected p-value <0.05)

**Table S4**: single group meta-analysis for the secondary analysis (principal and sensitivity analyses). HC: healthy controls, BPD: Borderline Personality Disorders. Results are cluster-wise corrected (uncorrected p-value < 0.001, cluster-wise corrected p-value <0.05)

|  |  |  |  | Centre of mass | | | Peak | | |  |  |
| --- | --- | --- | --- | --- | --- | --- | --- | --- | --- | --- | --- |
| Contrast | **Hemisphere** | **Region** | **BA** | **x** | **y** | **z** | **x** | **y** | **z** | **Peak ALE p-value** | **Volume (mm³)** |
| HC | L | Insula | 13 | -34.7 | 19.8 | 7.2 | -32 | 18 | 8 | 0.024 | 1744 |
|  | L | Inferior Frontal Gyrus | 45 |  |  |  | -34 | 24 | 6 | 0.023 |  |
|  | L | Inferior Frontal Gyrus | 45 |  |  |  | -46 | 22 | 2 | 0.017 |  |
|  | R | Insula | 13 | 35.1 | 20 | 5.4 | 32 | 20 | 8 | 0.026 | 1456 |
|  | R | Inferior Frontal Gyrus | 47 |  |  |  | 44 | 28 | 2 | 0.019 |  |
|  | R | Insula | 13 |  |  |  | 42 | 14 | 0 | 0.018 |  |
|  | L | Superior Parietal Lobule | 7 | -28.8 | -57.6 | 38.5 | -28 | -58 | 40 | 0.019 | 768 |
|  | L | Precuneus | 7 |  |  |  | -22 | -62 | 40 | 0.019 |  |
|  | L | Angular Gyrus | 39 |  |  |  | -36 | -56 | 34 | 0.017 |  |
|  | L | Medial Frontal Gyrus | 6 | -5 | 8.4 | 46.6 | -6 | 6 | 48 | 0.020 | 760 |
|  | L | Medial Frontal Gyrus | 32 |  |  |  | -2 | 10 | 44 | 0.020 |  |
| HC Emot | L | Inferior Frontal Gyrus | 45 | -38.4 | 21.6 | 4.6 | -36 | 24 | 6 | 0.017 | 896 |
|  | L | Inferior Frontal Gyrus | 45 |  |  |  | -42 | 20 | 4 | 0.017 |  |
|  | L | Insula | 13 |  |  |  | -32 | 20 | 4 | 0.017 |  |
|  | L | Amygdala |  | -23 | -5.9 | -9.5 | -22 | -6 | -10 | 0.022 | 768 |
|  | R | Inferior Frontal Gyrus | 47 | 42.9 | 24.1 | 1.6 | 44 | 28 | 2 | 0.019 | 672 |
|  | R | Insula | 13 |  |  |  | 42 | 14 | 2 | 0.015 |  |
|  | R | Inferior Frontal Gyrus | 47 |  |  |  | 42 | 22 | 2 | 0.014 |  |
| BPD | L | Insula | 13 | -31.6 | 15.6 | 7.6 | -32 | 16 | 8 | 0.027 | 992 |
| BPD Active | L | Insula | 13 | -31.5 | 16.3 | 7.1 | -32 | 18 | 6 | 0.023 | 712 |
| BPD Emot | R | Inferior Frontal Gyrus | 45 | 47.6 | 25.9 | 10.7 | 50 | 24 | 12 | 0.019 | 800 |

**Table S5:** Secondary analysis: significant clusters for difference of convergence between Healthy Controls (HC) and Borderline Personality Disorder Patients (BDP)*.* Results are cluster-wise corrected (corrected p-value <0.05)

|  |  |  |  | Centre of mass | | | Peak | | |  |  |
| --- | --- | --- | --- | --- | --- | --- | --- | --- | --- | --- | --- |
| Contrast | **Hemisphere** | **Region** | **BA** | **x** | **y** | **z** | **x** | **y** | **z** | **Peak ALE p-value** | **Volume (mm³)** |
| HC > BDP | R | Insula/Inferior Frontal Gyrus | 13 | 32.2 | 21.6 | 10.4 | 30 | 22 | 12 | 0.018 | 176 |

**Table S6:** Contrasts and studies used in the sensitivity analysis of the secondary analysis using the same contrasts in both the difference of convergence and convergence of difference analyses. HC: healthy controls; BPD: Borderline Personality Disorders*;* URE: Unresolved life events; RLE: Resolved life events; Inc-emo, Incongruent emotion; Cong-emo; Congruent emotion; Inc-neut: Incongruent neutral; Cong-neut; Congruent neutral; RepeatNeg: Repeat negative pictures; NovelNeg: Novel negative pictures; EMR: Episodic memory retrieval; BC: Baseline condition; Affective ToM: Affective theory of mind.

| **Study** | **BPD > HC** | **HC > BPD** | **HC** | **BPD** |
| --- | --- | --- | --- | --- |
| Aguilar-Ortiz, 2019 | 2-back vs baseline  1-back versus baseline | 2-back vs 1-back | 2-back vs baseline  2-back vs 1-back  1-back versus baseline | 2-back vs baseline  2-back vs 1-back  1-back versus baseline |
| Beblo, 2006 | URE vs RLE |  |  | URE vs RLE |
| Buchheim, 2008 | Monadic  Dyadic | Monadic  Dyadic | Monadic  Dyadic | Monadic  Dyadic |
| Frick, 2012 | Negative > Neutral  Positive > Neutral | Negative > Neutral  Positive > Neutral | Negative vs. Neutral  Positive > Neutral | Negative > Neutral  Positive > Neutral |
| Herbort, 2016 |  | Differences during gain and loss anticipation | Gain anticipation  Loss anticipation | Gain anticipation  Loss anticipation |
| Herpertz, 2001 | Negative > Neutral |  | Negative > Neutral | Negative > Neutral |
| Holtmann, 2013 | Fearful > Neutral  Incongruent > Congruent  Interaction emotion congruency:  (Inc-emo> Cong-emo) > (Inc-neut > Cong-neut) |  | Fearful > Neutral  Incongruent > Congruent  Interaction congruency by emotion | Fearful > Neutral  Incongruent > Congruent  Interaction congruency by emotion |
| Koenigsberg, Biol Psychiatry, 2009 | Distancing - Looking at negative pictures | Distancing - Looking at negative pictures | Distance > Look (negative pictures) | Distance > Look (negative pictures) |
| Koenigsberg, 2014 |  | RepeatNeg > NovelNeg | NovelNeg > RepeatNeg  RepeatNeg > NovelNeg | NovelNeg > RepeatNeg  RepeatNeg > NovelNeg |
| Kraus, 2010 | Trigger section | Emotional and cognitive reactions vs. Baseline |  | Trigger situation vs. Baseline  Emotional and cognitive reactions vs. Baseline |
| Lamers, 2019 | Negative – Neutral |  | Negative – Neutral | Negative – Neutral |
| Lang, 2012 |  | Up vs. maintain  Down vs. maintain | Up > Maintain  Down > Maintain | Up > Maintain  Down > Maintain |
| Mensebach, 2009 | EMR – BC  SMR - BC |  | EMR – BC  SMR - BC | EMR – BC  SMR - BC |
| Mier, 2013 | Affective ToM  Emotion recognition  Neutral face processing  Affective ToM > Emotion recognition | Affective ToM  Emotion recognition  Neutral face processing  Affective ToM > Emotion recognition | Affective ToM  Emotion recognition  Neutral face processing  Affective ToM > Emotion recognition | Affective ToM  Emotion recognition  Neutral face recognition |
| Minzenberg, 2007 | Fear minus Neutral  Neutral minus Fear  Anger minus Neutral  Neutral minus Anger | Fear minus Neutral  Neutral minus Fear  Anger minus Neutral | Fear minus Neutral  Neutral minus Fear  Anger minus Neutral  Neutral minus Anger | Fear minus Neutral  Neutral minus Fear  Anger minus Neutral |
| Mortensen, 2016 | Cue primes > Neutral primes | Cue primes > Neutral primes | Cue primes > Neutral primes | Cue primes > Neutral primes |
| Scherpiet, 2014 | Anticipation of negative stimuli > Anticipation of neutral stimuli  Anticipation of unknown stimuli > Anticipation of neutral stimuli  Anticipation of positive stimuli > Anticipation of neutral stimuli |  | Anticipation of negative stimuli > Anticipation of neutral stimuli  Anticipation of unknown stimuli > Anticipation of neutral stimuli  Anticipation of positive stimuli > Anticipation of neutral stimuli | Anticipation of negative stimuli > Anticipation of neutral stimuli  Anticipation of unknown stimuli > Anticipation of neutral stimuli  Anticipation of positive stimuli > Anticipation of neutral stimuli |
| Scherpiet, 2015 | Feel > Neutral  Think > Neutral | Feel > Think | Feel > Think  Feel > Neutral | Feel > Think  Feel > Neutral  Think > Neutral |
| Schmahl, 2006 | Fixed temperature Early Phase  Individual temperature Early Phase  Individual temperature Late Phase | Fixed temperature Early Phase  Individual temperature Early Phase  Individual temperature Late Phase | Fixed temperature  Individual temperature | Fixed temperature  Individual temperature |
| Schulze, 2011 | Regulation of negative emotions:  Decrease > Maintain  Increase > Maintain | Initial viewing phase:  Negative > Neutral  Regulation of negative emotions:  Decrease > Maintain  Increase > Maintain | Initial viewing phase:  Negative > Neutral  Regulation of negative emotions:  Decrease > Maintain  Increase > Maintain | Initial viewing phase:  Negative > Neutral  Regulation of negative emotions:  Decrease > Maintain  Increase > Maintain |
| Wingenfeld, 2009 |  | General negative words > Neutral words  Individual negative words > Neutral words | General negative words > Neutral words  Individual negative words > Neutral words |  |
| Winter, 2015 (neutral) | Positive - Neutral |  |  | Positive - Neutral |

**Table S7:** Secondary analysis sensitivity analysis using studies which have both the between group (i.e., HC > BPD and BPD > HC) and the single groups analyses and in which identical contrasts of interest were run for both type of analysis. Method 1: convergence of difference (uncorrected p-value < 0.001, cluster-wise corrected p-value <0.05); Method 2: difference of convergence (corrected p-value < 0.05). HC: healthy controls, BPD: Borderline Personality Disorders.

|  |  |  |  | |  | | Centre of mass | | | | | Peak | | | | | |  |  | |  |
| --- | --- | --- | --- | --- | --- | --- | --- | --- | --- | --- | --- | --- | --- | --- | --- | --- | --- | --- | --- | --- | --- |
| Method | **Contrast** | **Hemisphere** | **Region** | | **BA** | | **x** | | **y** | | **z** | **x** | | **y** | | **z** | | **Peak ALE p-value** | **Volume (mm³)** | |  |
| (1) | HC > BPD | L | Anterior Cingulate | | 24 | | -2.5 | | 30.6 | | 4.1 | -4 | | 36 | | 6 | | 0.014 | 680 | |  |
|  |  | L | Anterior Cingulate | | 24 | |  | |  | |  | 0 | | 24 | | 2 | | 0.014 |  | |  |
| (2) | HC > BDP | L | Inferior Frontal Gyrus | 13 | | -39.5 | | 21.3 | | 7.7 | | -40 | 22 | | 8 | | 0.019 | | | 112 | |

**S5. Supplementary figures**

**
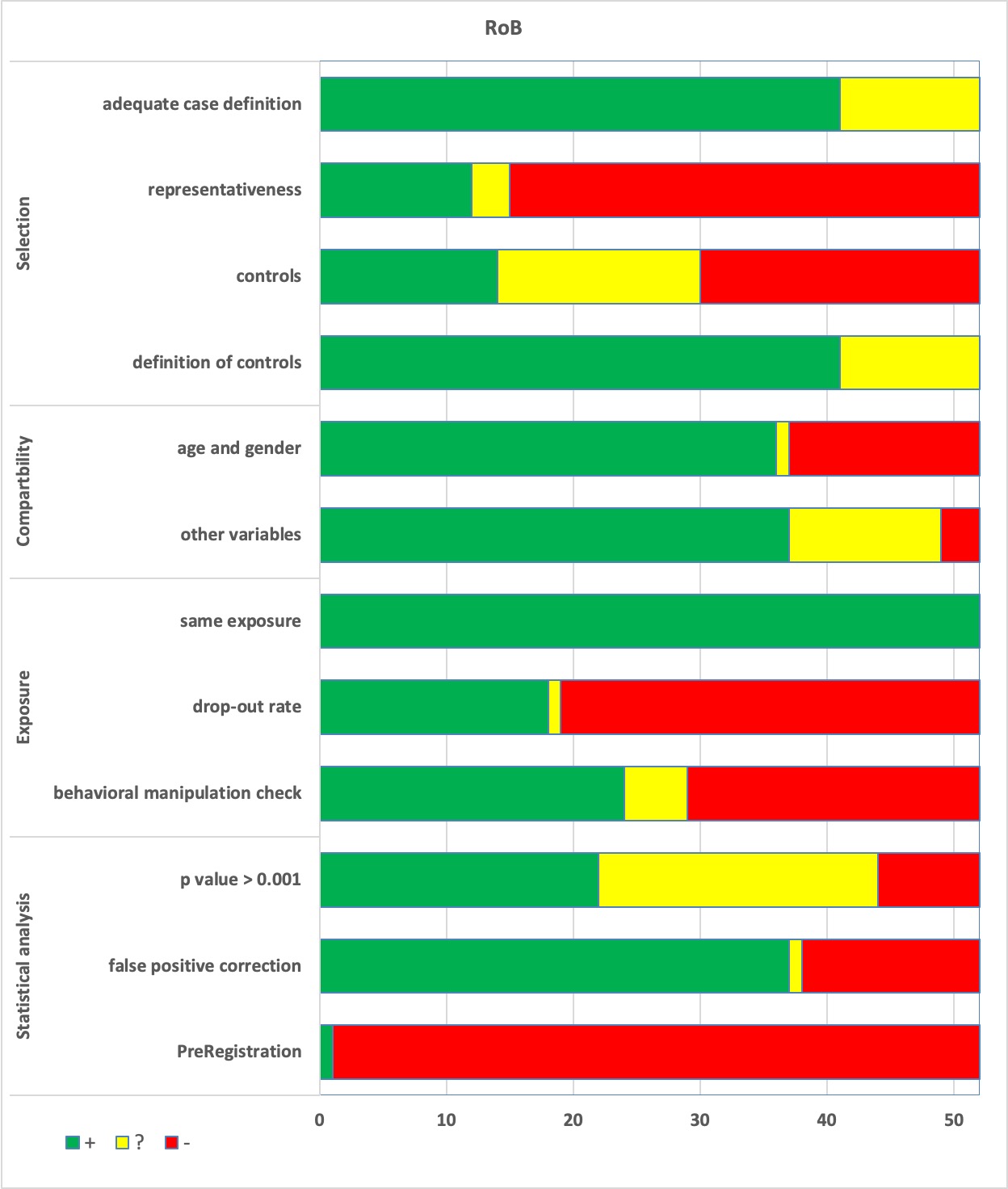
**

**Figure S1.** Risk of bias for the modified NOS-scale: + green color: low risk; - red color: high risk; ? yellow color: unclear


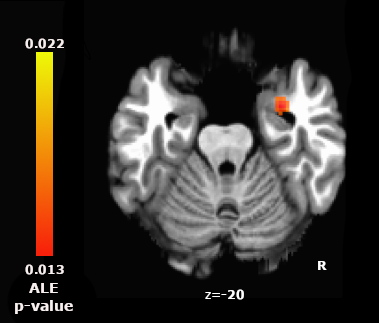


**Figure S2**: Sensitivity analysis for the primary analysis (convergence of difference) including studies with unmedicated patients only for the BPD > HC meta-analysis. Results are cluster-wise corrected (uncorrected p-value < 0.001, cluster-wise corrected p-value <0.05).


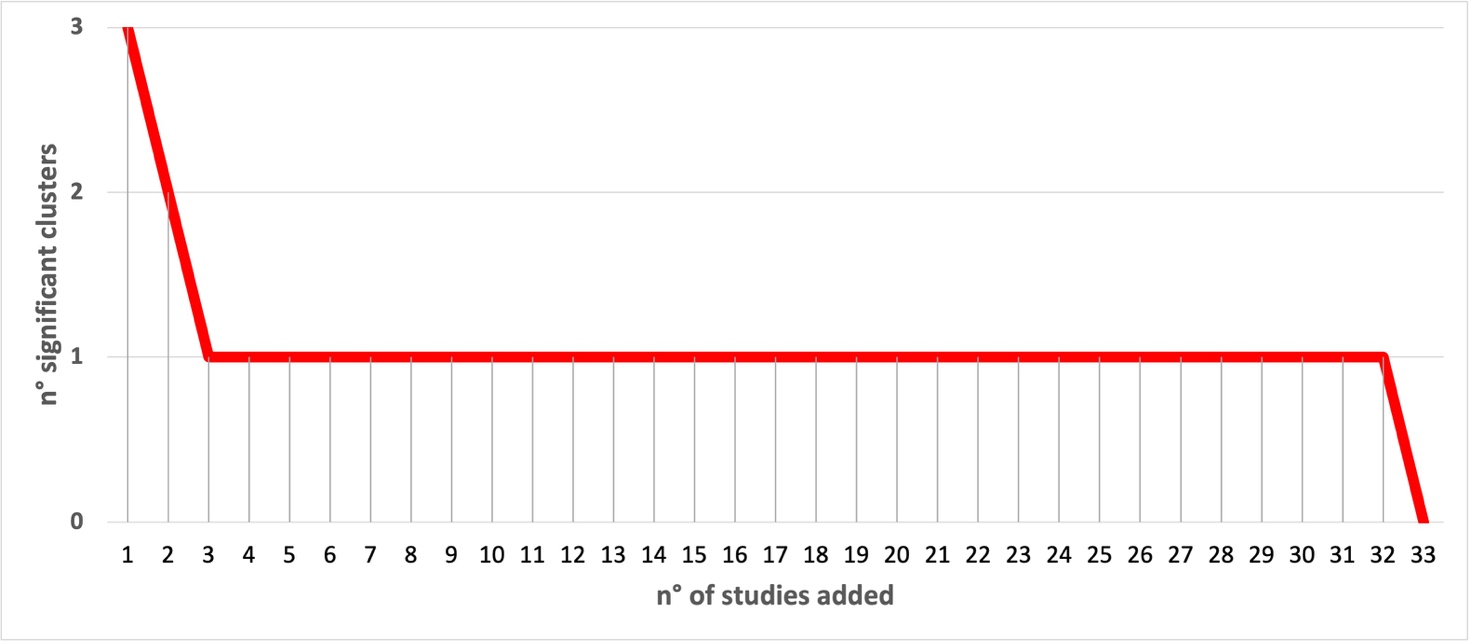


**Figure S3:** Effect of adding null studies to the meta-analysis on the significance of the 3 clusters found in the sensitivity analysis on emotion processing studies


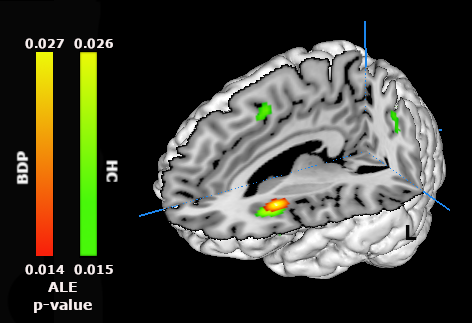


**Figure S4**: Secondary analysis (difference of convergence): convergence of activations for each single group separately: Borderline Patients (BDP – red colors) and Healthy Controls (HC – green colors). Results are cluster-wise corrected (uncorrected p-value < 0.001, cluster-wise corrected p-value <0.05).


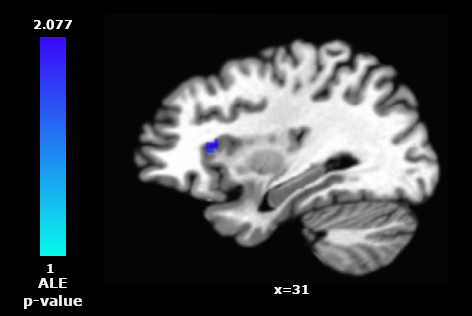


**Figure S5:** Secondary analysis (difference of convergence) for the contrast HC > BPD (corrected p-value < 0.05).


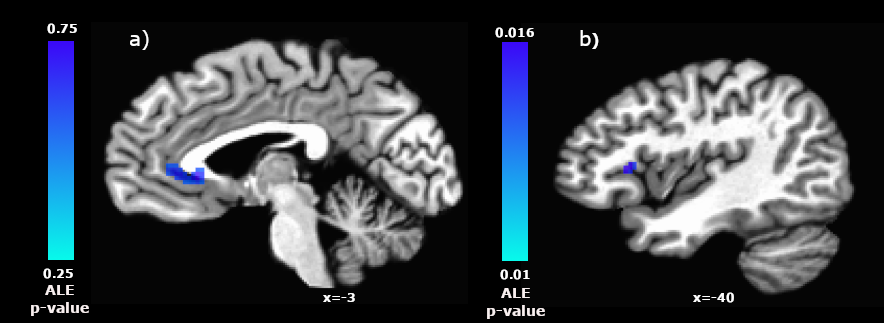


**Figure S6**. Sensitivity analysis for the secondary analysis (difference of convergence) using studies which have both the between group (i.e., HC > BPD and BPD > HC) and the single groups analyses and in which identical contrasts of interest were run for both type of analysis. a): convergence of difference (uncorrected p-value < 0.001, cluster-wise corrected p-value <0.05); b) difference of convergence (corrected p-value < 0.05). Blue colors indicate the HC > BDP contrasts for both the methods.

**S6 Supplementary references**

1 Eklund, A., Nichols, T. E. & Knutsson, H. Cluster failure: Why fMRI inferences for spatial extent have inflated false-positive rates. *Proc Natl Acad Sci U S A* **113**, 7900-7905, doi:10.1073/pnas.1602413113 (2016).

2 Gentili, C., Messerotti Benvenuti, S., Lettieri, G., Costa, C. & Cecchetti, L. ROI and phobias: The effect of ROI approach on an ALE meta-analysis of specific phobias. *Hum Brain Mapp* **40**, 1814-1828, doi:10.1002/hbm.24492 (2019).

3 Aguilar-Ortiz, S. *et al.* Evidence for default mode network dysfunction in borderline personality disorder. *Psychological Medicine*, 1-9, doi:<https://dx.doi.org/10.1017/S0033291719001880> (2019).

4 Beblo, T. *et al.* Functional MRI correlates of the recall of unresolved life events in borderline personality disorder. *Psychol Med* **36**, 845-856, doi:10.1017/s0033291706007227 (2006).

5 Beeney, J. E., Hallquist, M. N., Ellison, W. D. & Levy, K. N. Self-other disturbance in borderline personality disorder: Neural, self-report, and performance-based evidence. *Personal Disord* **7**, 28-39, doi:10.1037/per0000127 (2016).

6 Brown, R. C. *et al.* Differential Neural Processing of Social Exclusion and Inclusion in Adolescents with Non-Suicidal Self-Injury and Young Adults with Borderline Personality Disorder. *Front Psychiatry* **8**, 267, doi:10.3389/fpsyt.2017.00267 (2017).

7 Buchheim, A. *et al.* Neural correlates of attachment trauma in borderline personality disorder: a functional magnetic resonance imaging study. *Psychiatry Res* **163**, 223-235, doi:10.1016/j.pscychresns.2007.07.001 (2008).

8 Cullen, K. R. *et al.* Brain activation in response to overt and covert fear and happy faces in women with borderline personality disorder. *Brain Imaging Behav* **10**, 319-331, doi:10.1007/s11682-015-9406-4 (2016).

9 Doell, K. C. *et al.* Atypical processing of social anticipation and feedback in borderline personality disorder. *NeuroImage Clinical* **25**, 102126, doi:<https://dx.doi.org/10.1016/j.nicl.2019.102126> (2020).

10 Domsalla, M. *et al.* Cerebral processing of social rejection in patients with borderline personality disorder. *Soc Cogn Affect Neurosci* **9**, 1789-1797, doi:10.1093/scan/nst176 (2014).

11 Dudas, R. B. *et al.* Amygdala and dlPFC abnormalities, with aberrant connectivity and habituation in response to emotional stimuli in females with BPD. *J Affect Disord* **208**, 460-466, doi:10.1016/j.jad.2016.10.043 (2017).

12 Fertuck, E. A. *et al.* Trustworthiness appraisal deficits in borderline personality disorder are associated with prefrontal cortex, not amygdala, impairment. *Neuroimage Clin* **21**, 101616, doi:10.1016/j.nicl.2018.101616 (2019).

13 Frick, C. *et al.* Hypersensitivity in borderline personality disorder during mindreading. *PLoS One* **7**, e41650, doi:10.1371/journal.pone.0041650 (2012).

14 Gottlich, M. *et al.* Neural basis of shame and guilt experience in women with borderline personality disorder. *European Archives of Psychiatry & Clinical Neuroscience* **07**, 07, doi:<https://dx.doi.org/10.1007/s00406-020-01132-z> (2020).

15 Guitart-Masip, M. *et al.* Neural correlates of impaired emotional discrimination in borderline personality disorder: an fMRI study. *Prog Neuropsychopharmacol Biol Psychiatry* **33**, 1537-1545, doi:10.1016/j.pnpbp.2009.08.022 (2009).

16 Hazlett, E. A. *et al.* Potentiated amygdala response to repeated emotional pictures in borderline personality disorder. *Biol Psychiatry* **72**, 448-456, doi:10.1016/j.biopsych.2012.03.027 (2012).

17 Herpertz, S. C. *et al.* Evidence of abnormal amygdala functioning in borderline personality disorder: a functional MRI study. *Biol Psychiatry* **50**, 292-298, doi:10.1016/s0006-3223(01)01075-7 (2001).

18 Herpertz, S. C. *et al.* Brain Mechanisms Underlying Reactive Aggression in Borderline Personality Disorder-Sex Matters. *Biol Psychiatry* **82**, 257-266, doi:10.1016/j.biopsych.2017.02.1175 (2017).

19 Holtmann, J. *et al.* Trait anxiety modulates fronto-limbic processing of emotional interference in borderline personality disorder. *Front Hum Neurosci* **7**, 54, doi:10.3389/fnhum.2013.00054 (2013).

20 Homan, P., Reddan, M. C., Brosch, T., Koenigsberg, H. W. & Schiller, D. Aberrant link between empathy and social attribution style in borderline personality disorder. *J Psychiatr Res* **94**, 163-171, doi:10.1016/j.jpsychires.2017.07.012 (2017).

21 King-Casas, B. *et al.* The rupture and repair of cooperation in borderline personality disorder. *Science* **321**, 806-810, doi:10.1126/science.1156902 (2008).

22 Koenigsberg, H. W. *et al.* Neural correlates of emotion processing in borderline personality disorder. *Psychiatry Res* **172**, 192-199, doi:10.1016/j.pscychresns.2008.07.010 (2009).

23 Krauch, M. *et al.* Heightened Salience of Anger and Aggression in Female Adolescents With Borderline Personality Disorder-A Script-Based fMRI Study. *Front Behav Neurosci* **12**, 57, doi:10.3389/fnbeh.2018.00057 (2018).

24 Kraus, A. *et al.* Script-driven imagery of self-injurious behavior in patients with borderline personality disorder: a pilot FMRI study. *Acta Psychiatr Scand* **121**, 41-51, doi:10.1111/j.1600-0447.2009.01417.x (2010).

25 Krause-Utz, A. *et al.* Reduced amygdala reactivity and impaired working memory during dissociation in borderline personality disorder. *Eur Arch Psychiatry Clin Neurosci* **268**, 401-415, doi:10.1007/s00406-017-0806-x (2018).

26 Lang, S. *et al.* Cognitive reappraisal in trauma-exposed women with borderline personality disorder. *Neuroimage* **59**, 1727-1734, doi:10.1016/j.neuroimage.2011.08.061 (2012).

27 Malejko, K. *et al.* Neural Correlates of Social Inclusion in Borderline Personality Disorder. *Front Psychiatry* **9**, 653, doi:10.3389/fpsyt.2018.00653 (2018).

28 Malejko, K. *et al.* Somatosensory Stimulus Intensity Encoding in Borderline Personality Disorder. *Front Psychol* **9**, 1853, doi:10.3389/fpsyg.2018.01853 (2018).

29 Mensebach, C. *et al.* Neural correlates of episodic and semantic memory retrieval in borderline personality disorder: an fMRI study. *Psychiatry Res* **171**, 94-105, doi:10.1016/j.pscychresns.2008.02.006 (2009).

30 Mier, D. *et al.* Neuronal correlates of social cognition in borderline personality disorder. *Soc Cogn Affect Neurosci* **8**, 531-537, doi:10.1093/scan/nss028 (2013).

31 Minzenberg, M. J., Fan, J., New, A. S., Tang, C. Y. & Siever, L. J. Fronto-limbic dysfunction in response to facial emotion in borderline personality disorder: an event-related fMRI study. *Psychiatry Res* **155**, 231-243, doi:10.1016/j.pscychresns.2007.03.006 (2007).

32 Mortensen, J. A., Evensmoen, H. R., Klensmeden, G. & Haberg, A. K. Outcome Uncertainty and Brain Activity Aberrance in the Insula and Anterior Cingulate Cortex Are Associated with Dysfunctional Impulsivity in Borderline Personality Disorder. *Front Hum Neurosci* **10**, 207, doi:10.3389/fnhum.2016.00207 (2016).

33 Peters, J. R., Chester, D. S., Walsh, E. C., DeWall, C. N. & Baer, R. A. The rewarding nature of provocation-focused rumination in women with borderline personality disorder: a preliminary fMRI investigation. *Borderline Personal Disord Emot Dysregul* **5**, 1, doi:10.1186/s40479-018-0079-7 (2018).

34 Scherpiet, S. *et al.* Altered emotion processing circuits during the anticipation of emotional stimuli in women with borderline personality disorder. *Eur Arch Psychiatry Clin Neurosci* **264**, 45-60, doi:10.1007/s00406-013-0444-x (2014).

35 Schmahl, C. *et al.* Neural correlates of antinociception in borderline personality disorder. *Arch Gen Psychiatry* **63**, 659-667, doi:10.1001/archpsyc.63.6.659 (2006).

36 Schnell, K., Dietrich, T., Schnitker, R., Daumann, J. & Herpertz, S. C. Processing of autobiographical memory retrieval cues in borderline personality disorder. *J Affect Disord* **97**, 253-259, doi:10.1016/j.jad.2006.05.035 (2007).

37 Schnell, K. & Herpertz, S. C. Effects of dialectic-behavioral-therapy on the neural correlates of affective hyperarousal in borderline personality disorder. *J Psychiatr Res* **41**, 837-847, doi:10.1016/j.jpsychires.2006.08.011 (2007).

38 Silbersweig, D. *et al.* Failure of frontolimbic inhibitory function in the context of negative emotion in borderline personality disorder. *Am J Psychiatry* **164**, 1832-1841, doi:10.1176/appi.ajp.2007.06010126 (2007).

39 Sosic-Vasic, Z. *et al.* Mirror neuron activations in encoding of psychic pain in borderline personality disorder. *Neuroimage Clin* **22**, 101737, doi:10.1016/j.nicl.2019.101737 (2019).

40 van Schie, C. C., Chiu, C. D., Rombouts, S., Heiser, W. J. & Elzinga, B. M. Stuck in a negative me: fMRI study on the role of disturbed self-views in social feedback processing in borderline personality disorder. *Psychological Medicine* **50**, 625-635, doi:<https://dx.doi.org/10.1017/S0033291719000448> (2020).

41 van Zutphen, L. *et al.* Impulse control under emotion processing: an fMRI investigation in borderline personality disorder compared to non-patients and cluster-C personality disorder patients. *Brain Imaging Behav*, doi:10.1007/s11682-019-00161-0 (2019).

42 Wingenfeld, K. *et al.* Neural correlates of the individual emotional Stroop in borderline personality disorder. *Psychoneuroendocrinology* **34**, 571-586, doi:10.1016/j.psyneuen.2008.10.024 (2009).

43 Winter, D. *et al.* Dissociation in borderline personality disorder: Disturbed cognitive and emotional inhibition and its neural correlates. *Psychiatry Res* **233**, 339-351, doi:10.1016/j.pscychresns.2015.05.018 (2015).

44 Wrege, J. S. *et al.* Negative affect moderates the effect of social rejection on frontal and anterior cingulate cortex activation in borderline personality disorder. *Cogn Affect Behav Neurosci*, doi:10.3758/s13415-019-00716-0 (2019).

45 Herbort, M. C. *et al.* A negative relationship between ventral striatal loss anticipation response and impulsivity in borderline personality disorder. *Neuroimage Clin* **12**, 724-736, doi:10.1016/j.nicl.2016.08.011 (2016).

46 Koenigsberg, H. W. *et al.* The neural correlates of anomalous habituation to negative emotional pictures in borderline and avoidant personality disorder patients. *Am J Psychiatry* **171**, 82-90, doi:10.1176/appi.ajp.2013.13070852 (2014).

47 Nicol, K., Pope, M., Romaniuk, L. & Hall, J. Childhood trauma, midbrain activation and psychotic symptoms in borderline personality disorder. *Transl Psychiatry* **5**, e559, doi:10.1038/tp.2015.53 (2015).

48 Scherpiet, S. *et al.* Reduced neural differentiation between self-referential cognitive and emotional processes in women with borderline personality disorder. *Psychiatry Res* **233**, 314-323, doi:10.1016/j.pscychresns.2015.05.008 (2015).

49 Bertsch, K. *et al.* Out of control? Acting out anger is associated with deficient prefrontal emotional action control in male patients with borderline personality disorder. *Neuropharmacology* **156**, 107463, doi:10.1016/j.neuropharm.2018.12.010 (2019).

50 Koenigsberg, H. W. *et al.* Neural correlates of the use of psychological distancing to regulate responses to negative social cues: a study of patients with borderline personality disorder. *Biol Psychiatry* **66**, 854-863, doi:10.1016/j.biopsych.2009.06.010 (2009).

51 Lamers, A. *et al.* Nonacceptance of negative emotions in women with borderline personality disorder: association with neuroactivity of the dorsal striatum. *J Psychiatry Neurosci* **44**, 303-312, doi:10.1503/jpn.180077 (2019).

52 Niedtfeld, I. *et al.* Affect regulation and pain in borderline personality disorder: a possible link to the understanding of self-injury. *Biol Psychiatry* **68**, 383-391, doi:10.1016/j.biopsych.2010.04.015 (2010).

53 Schulze, L. *et al.* Neuronal correlates of cognitive reappraisal in borderline patients with affective instability. *Biol Psychiatry* **69**, 564-573, doi:10.1016/j.biopsych.2010.10.025 (2011).

54 van Zutphen, L. *et al.* Always on guard: emotion regulation in women with borderline personality disorder compared to nonpatient controls and patients with cluster-C personality disorder. *Journal of Psychiatry & Neuroscience* **43**, 37-47 (2018).

55 Cox, R. W., Chen, G., Glen, D. R., Reynolds, R. C. & Taylor, P. A. fMRI clustering and false-positive rates. *Proc. Natl. Acad. Sci. U.S.A.* **114**, E3370-E3371, doi:10.1073/pnas.1614961114 (2017).
